# μMESH-Enabled Sustained Delivery of Molecular and Nanoformulated Drugs for Glioblastoma Treatment

**DOI:** 10.1021/acsnano.3c01574

**Published:** 2023-06-28

**Authors:** Daniele Di Mascolo, Irene Guerriero, Cristiano Pesce, Raffaele Spanò, Anna Lisa Palange, Paolo Decuzzi

**Affiliations:** † Laboratory of Nanotechnology for Precision Medicine, Fondazione Istituto Italiano di Tecnologia, 16163 Genoa, Italy; ‡ Department of Electrical and Information Engineering, Politecnico di Bari, 70126 Bari, Italy; § Department of Informatics, Bioengineering, Robotics and System Engineering, Università di Genova, 16145 Genova, Italy; ∥ Department of Pharmaceutical and Pharmacological Sciences, University of Padua, 35122 Padova, Italy

**Keywords:** locoregional therapy, high-grade glioma, drug delivery, nanomedicine, taxanes

## Abstract

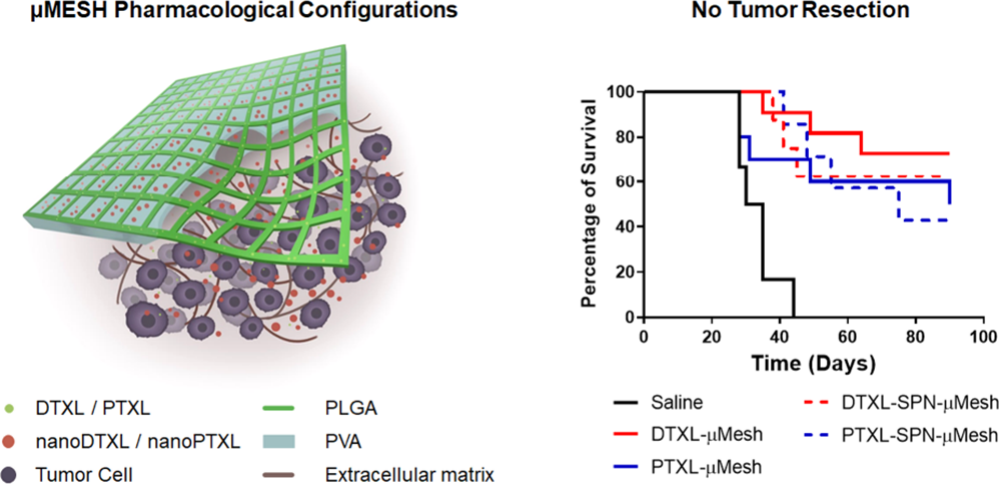

Modest tissue penetrance, nonuniform distribution, and suboptimal release of drugs limit the potential of intracranial therapies against glioblastoma. Here, a conformable polymeric implant, μMESH, is realized by intercalating a micronetwork of 3 × 5 μm poly(lactic-*co*-glycolic acid) (PLGA) edges over arrays of 20 × 20 μm polyvinyl alcohol (PVA) pillars for the sustained delivery of potent chemotherapeutic molecules, docetaxel (DTXL) and paclitaxel (PTXL). Four different μMESH configurations were engineered by encapsulating DTXL or PTXL within the PLGA micronetwork and nanoformulated DTXL (nanoDTXL) or PTXL (nanoPTXL) within the PVA microlayer. All four μMESH configurations provided sustained drug release for at least 150 days. However, while a burst release of up to 80% of nanoPTXL/nanoDTXL was documented within the first 4 days, molecular DTXL and PTXL were released more slowly from μMESH. Upon incubation with U87-MG cell spheroids, DTXL-μMESH was associated with the lowest lethal drug dose, followed by nanoDTXL-μMESH, PTXL-μMESH, and nanoPTXL-μMESH. In orthotopic models of glioblastoma, μMESH was peritumorally deposited at 15 days post-cell inoculation and tumor proliferation was monitored via bioluminescence imaging. The overall animal survival increased from ∼30 days of the untreated controls to 75 days for nanoPTXL-μMESH and 90 days for PTXL-μMESH. For the DTXL groups, the overall survival could not be defined as 80% and 60% of the animals treated with DTXL-μMESH and nanoDTXL-μMESH were still alive at 90 days, respectively. These results suggest that the sustained delivery of potent drugs properly encapsulated in conformable polymeric implants could halt the proliferation of aggressive brain tumors.

Despite the drop in cancer death rates over the last three decades, which has been mostly dictated by pervasive prevention and screening campaigns as well as more effective targeted therapies, glioblastoma (GBM) continues to be the less curable form of cancer with a 5-year survival rate of only 5%.^1,2^ The standard of care for GBM has not changed over the past 20 years, as it still relies on maximal safe resection of the malignant mass followed by adjuvant radiochemotherapy: the Stupp’s protocol.^3^ This complex and expensive treatment provides only a modest improvement in life expectancy and various degrees of therapy-induced complications, including the deterioration of physical, emotional, and social functions.^4,5^ Perhaps even more importantly, there is no standard of care in the event of a disease relapse, which occurs in ∼90% of the patients with the formation of secondary masses typically within 1 to 2 cm from the original tumor.^6^ Such a dismal prognosis mainly draws from two unique features of GBM: anatomical barriers, whereby the access to the diseased tissue of blood-borne agents is tremendously impaired by the presence of the blood–brain barrier (BBB) and a thick hyaluronan-rich extracellular matrix (ECM),^7−9^ and biological heterogeneity, resulting from the rapid and unbounded proliferation of malignant cells surrounded by a continuously morphing tumor microenvironment (TME) enriched by different subsets of immune and glioblastoma stem cells.^10−12^ In this context, drug delivery systems for locoregional therapies are expected to have unique advantages over systemic approaches as they would bypass the BBB and fully exploit the fact that the brain is a “closed system” in which therapeutic agents could achieve long half-lives.

Following this line of thought, already in the late ‘90s, the biodegradable wafer Gliadel, encapsulating carmustine in a 14 × 1 mm rigid disc, was developed for direct deposition within the resected tumor cavity.^13^ However, Gliadel has never shown any clear survival benefit over the standard of care, mostly because of the rapid drug release (<6 days), modest drug potency (IC_50_ ∼ 10 μM), and poor tissue integration.^13,14^ The convection-enhanced infusion of molecules, antibodies, and nanomedicines has also been proposed and tested clinically as a strategy to bypass the BBB and push therapeutic agents deep into the malignant tissue via an externally applied counterpressure.^15,16^ Despite the increased delivery doses and longer release profiles, this approach has led to contradictory results and no significant clinical success, largely because of pressure-induced brain tissue damage, edema, and unpredictable distribution of the infused agents.^17^*In situ* forming implants, based on the intracranial injection of polymeric mixtures, continue to be developed by several laboratories.^18^ However, their archetypal model OncoGel, which is based on a PEG–poly(lactic-*co*-glycolic acid) (PLGA) block copolymer, failed to demonstrate survival improvements in GBM and esophageal cancer.^19^ Within the last 20 years, only the tumor-treating-field (TTF) approach, based on the quasi-continuous exposure (>18 h/day for several months) of brain cells to low-intensity alternating electric fields, has shown some improvement in survival (<5 months).^20^ However, recent studies are highlighting the importance and limitations of patient compliance for this therapeutic approach.^21^

In short, limited drug penetration and distribution throughout the tumor, local reactions due to mechanical mismatch between the implanted devices and the surrounding tissue, suboptimal release profiles, and importantly, selection of payloads with modest cytotoxic potential have limited the efficacy of locoregional therapies. In this study, four different therapeutic configurations of the mechanically conformable, biodegradable, and compartmentalized implant μMESH^22^ have been realized, thoroughly characterized for their physicochemical and pharmacological properties, and preclinically validated in orthotopic murine models of glioblastoma. Two taxanes have been selected as anticancer agents,^23^ namely, docetaxel (DTXL) and paclitaxel (PTXL), and have been loaded into μMESH either in their native molecular form or as nanomedicines, upon encapsulation into conventional spherical polymeric nanoparticles.

## Results and Discussion

Cancer therapy has been radically improved in the past decade, especially with recent approaches such as immunotherapy. Still, some tumors did not benefit from such development, and among them, GBM remains most likely the most challenging one. The major obstacles limiting the efficacy of common therapeutics are the presence of the less penetrable biological barrier (i.e., the BBB), the significant heterogeneity between cancer cells even in the same patient, and the invasiveness of this type of tumor. Nanomedicine-based locoregional therapy could be a key to unlocking the full potential of the therapy, but more information must be acquired on some critical parameters: the effect of the solvents on drug solubility in device fabrication; the interaction between drug molecules and the materials their carriers are made of, and the resulting release profile. All these parameters were analyzed from a physicochemical, pharmacological, and biological point of view and used to tune μMESH-based formulations, entrapping drug molecules or drug-loaded nanoparticles, optimizing their efficacy.

### Geometrical Characterization of μMESH

**Figure 1:**
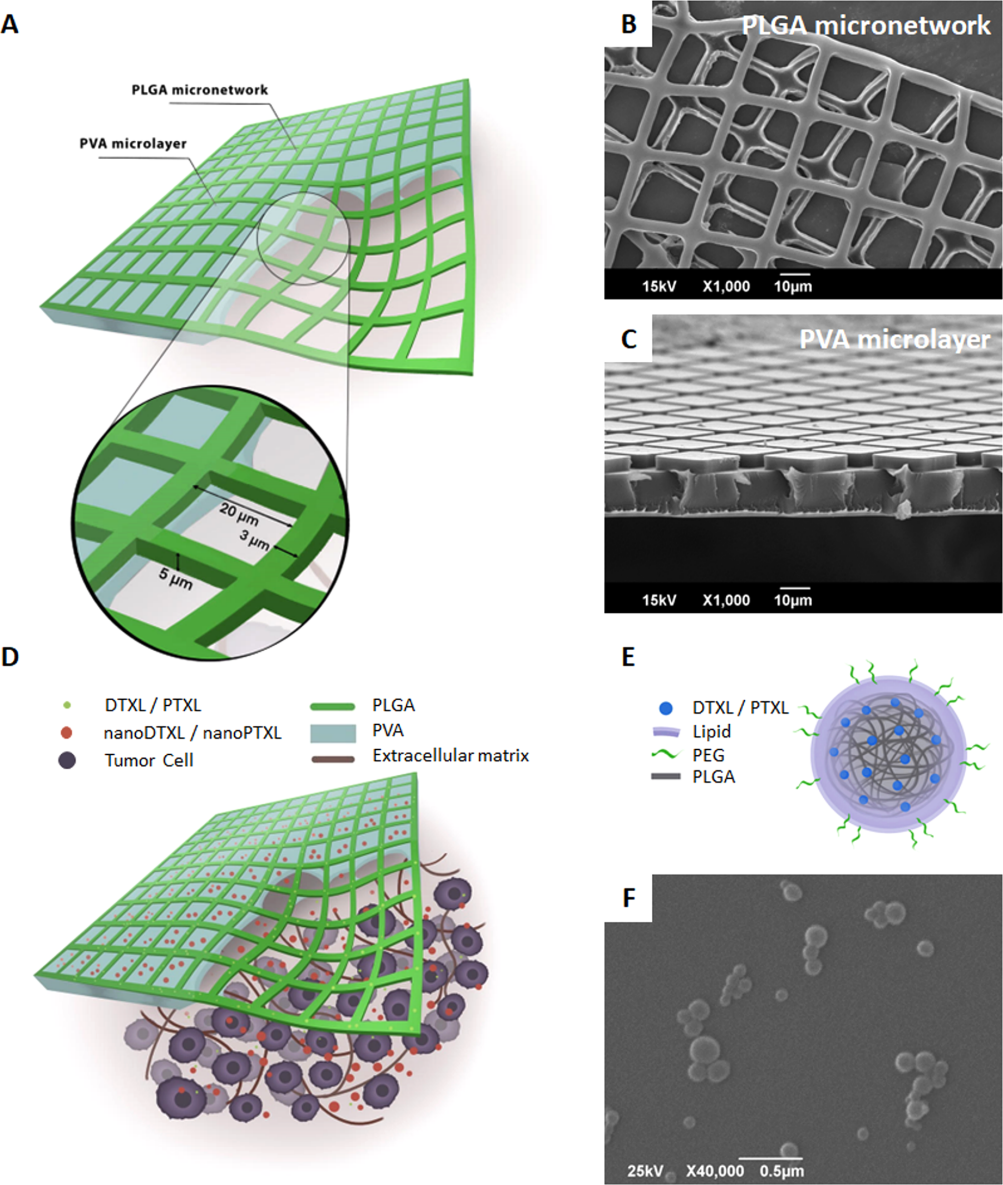
μMESH geometrical and pharmacological features. (A) μMESH schematic and dual-compartment architecture, including the PLGA micronetwork (green lines) and the PVA microlayer (azure layer). (B) Scanning electron microscopy of the PLGA micronetwork–hydrophobic compartment. (C) Scanning electron microscopy of the PVA microlayer–hydrophilic compartment. (D) μMESH deposition over the malignant tissue and progressive release of its payloads, including free DTXL or PTXL molecules (green dots), dispersed within the PLGA micronetwork, or nanoformulated DTXL or PTXL (nanoDTXL or nanoPTXL, red dots) incorporated in the PVA microlayer. (E) Spherical nanoparticles carrying DTXL (nanoDTXL) or PTXL (nanoPTXL) in a PLGA core are stabilized by a lipid monolayer. (F) SEM images of the spherical nanoparticles carrying DTXL or PTXL.

μMESH is a dual-compartment drug delivery system comprising a polyvinyl alcohol (PVA) microlayer supporting a PLGA micronetwork (Figure 1A–C). μMESH is realized through a top-down soft lithography fabrication approach, previously described by the authors,^22^ which allows for the precise control of all the geometrical attributes of the PVA microlayer and PLGA micronetwork. For the specific configuration considered in this work, μMESH is constituted by a regular network of PLGA microscopic edges with a 3 × 5 μm rectangular cross-section (width × height) delineating 20 × 20 μm square openings (Figure 1A–C). The network of microscopic PLGA edges, PLGA micronetwork (Figure 1B), lays and intercalates precisely with a regular matrix of 20 × 20 μm PVA square pillars, PVA microlayer (Figure 1C), presenting a height of 5 μm and a pitch distance of 3 μm. The overall thickness of the PVA microlayer is ∼20 μm. Although the geometrical attributes of μMESH can be modified by finely tuning the lithographic steps in the fabrication process, the current configuration offers an edge:opening ratio (3:20) much smaller than 1 to ensure mechanical flexibility (Figure 1B):^22^ the “fishing net”-like geometry and the deformability are two distinctive properties of μMESH.

The PLGA micronetwork and PVA microlayer can carry a broad variety of therapeutic agents. However, while the former would typically entrap hydrophobic compounds, the latter can be readily loaded with water-soluble agents. In this work, four different μMESH configurations were realized and characterized depending on the loading compartment for two highly potent cytotoxic molecules: docetaxel (DTXL) and paclitaxel (PTXL) (Figure 1D). In one case, free DTXL or PTXL molecules were uniformly dispersed within the edges of the PLGA micronetwork leading to DTXL-μMESH or PTXL-μMESH, whereas in another case, spherical polymeric nanoparticles carrying DTXL or PTXL (nanoDTXL or nanoPTXL) were encapsulated within the PVA microlayer leading to nanoDTXL-μMESH or nanoPTXL-μMESH. As detailed in the Materials and Methods and Supporting Information, the nanomedicines were obtained via a sonication/evaporation method^24,25^ returning particles with a spherical shape of around 200 nm whose PLGA core was stabilized by a lipid monolayer (Figure 1E,F).

### Pharmacological Characterizations of Drug-Loaded μMESH

**Figure 2:**
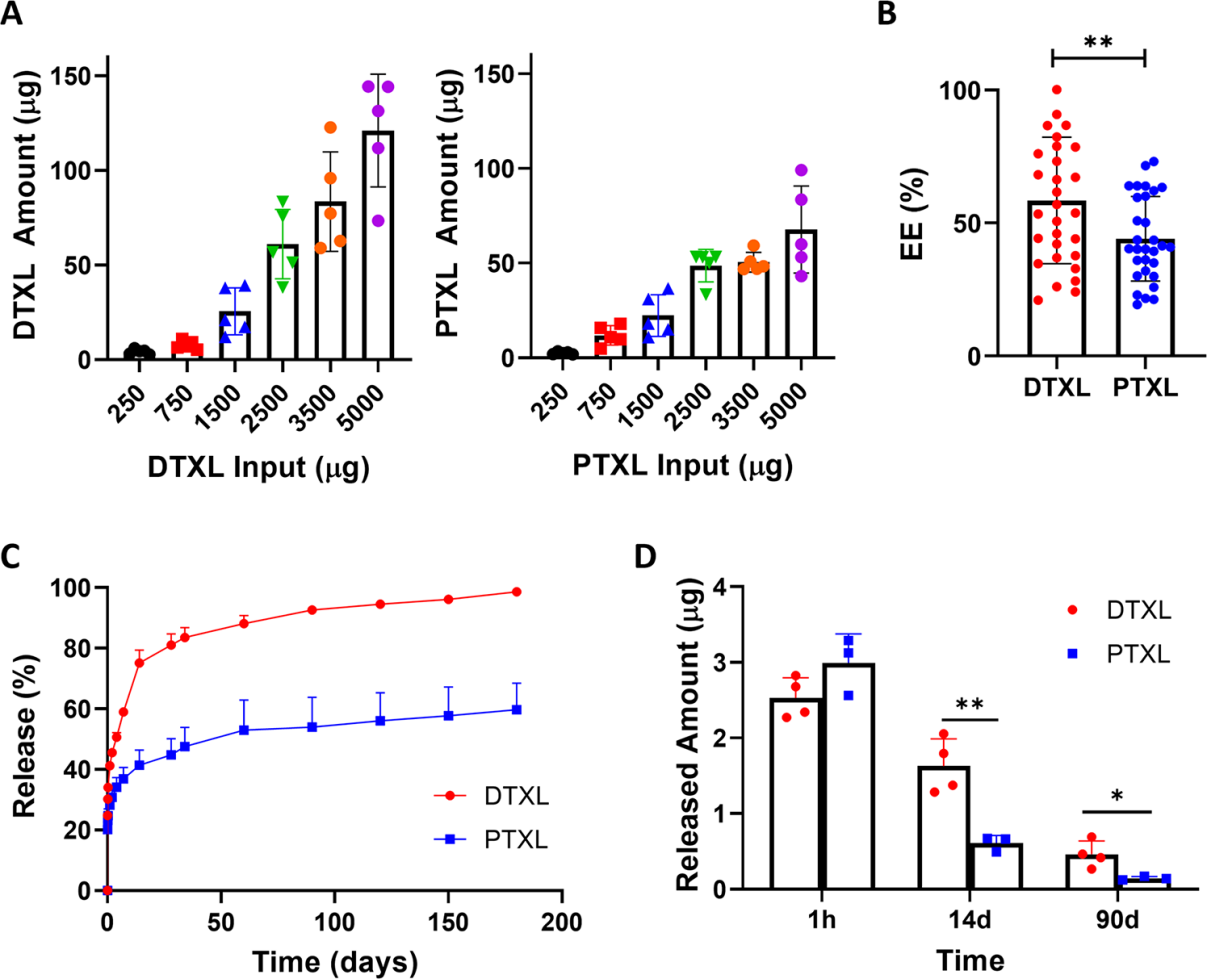
Pharmacological characterization of DTXL and PTXL loaded in μMESH. (A) Loaded amounts for DTXL (left) and PTXL (right), measured in 5 × 5 mm μMESH versus drug amounts input in full-sized 27.5 × 27.5 mm μMESH. (B) Encapsulation efficiency (EE) for all the tested DTXL- and PTXL-μMESH configurations. (*p* = 0.0077). (C) Drug release profiles from DTXL- and PTXL-μMESH over a 180 day observation period. (D) Amount of drug released from DTXL- and PTXL-μMESH at 1 h, 14 days, and 90 days (h: hour; d: day) (*, *p* = 0.03; **, *p* = 0.006).

For DXTL-μMESH and PTXL-μMESH, different input amounts of the corresponding anticancer drugs, namely, 250, 750, 1500, 2500, 3500, and 5000 μg, were dissolved in acetonitrile together with PLGA and carefully spread to fill the ridges in the PVA microlayer. Figure 2A presents the amounts of DTXL (left) and PTXL (right) loaded into μMESH for different drug inputs. There is a quasi-linear relationship between the input and loaded amounts with correlation coefficients of *R*^2^ = 0.86 and *R*^2^ ∼ 0.80 for DTXL and PTXL, respectively (Supporting Figure 1A). In general, free DTXL was loaded more efficiently than PTXL in the μMESH edges. For 5000 and 3500 μg of drug input, DTXL-μMESH returned loadings of 121.0 ± 29.8 and 83.6 ± 26.3 μg with an encapsulation efficiency (EE) of ∼70%, respectively. For similar input amounts, PTXL-μMESH returned loadings of 67.8 ± 23 and 50.5 ± 5.2 μg with an encapsulation efficiency of ∼40%. The overall EE calculated across all tested input amounts, ranging from 250 to 5000 μg, was significantly larger for DTXL-μMESH (58.43 ± 23.73) than for PTXL-μMESH (44.04 ± 15.87) (*p* = 0.0077) (Figure 2B and Supporting Figure 1B).

It is here important to highlight that the loaded drug amounts (Figure 2A) were measured by processing multiple (*n* = 5) 5 × 5 mm pieces of μMESH, taken from different locations (from one border to the other) out of a full-sized 27.5 × 27.5 mm μMESH. Therefore, the corresponding loading amounts for the full-sized μMESH would be readily computed by multiplying by 30 times the loading associated with each of the 5 × 5 mm pieces of μMESH. Also, this allowed the authors to confirm that the drug molecules were quite uniformly distributed within the PLGA edges of μMESH (Supporting Figure 2). Incidentally, a full-sized μMESH, whose dimensions are comparable to the average characteristic size of a human glioblastoma, could carry up to ∼4 mg of DTXL in the current configuration (∼200 mg/kg for a 20 g animal). Despite the similar chemical structure and molecular weight of the two drugs, DTXL appeared to interact more effectively with PLGA than PTXL. Indeed, further analyses revealed that PTXL and DTXL have different solubilities in acetonitrile, with the former being almost 10 times more soluble than the latter (46.1 ± 1.9 mg/mL for PTXL; 6.2 ± 0.9 mg/mL for DTXL) (Supporting Information). Therefore, especially at the higher concentrations in acetonitrile (i.e., 50 mg/mL), DTXL is expected to appear in the form of drug crystals, whereas PTXL would still be in its molecular form (Supporting Figure 3).^26^ Importantly, regardless of the input amounts, the DTXL solutions were easily and uniformly spread within the ridges of the PVA microlayer, thus resulting in higher loaded amounts than those for PTXL.

Following the loading characterization, the release of DTXL and PTXL from μMESH was measured as the cumulative drug release over time and expressed as the percentage of the total loaded drug amount. Figure 2C shows the drug release from DTXL-μMESH (red curve) and PTXL-μMESH (blue curve), realized with an initial drug input of ∼1000 μg and returning a loaded drug amount of ∼15 μg, corresponding to the dose used for the *in vivo* experiments (0.75 mg/kg for a 20 g animal).^22^ Note that under these conditions only a modest formation of DTXL crystals was observed (Supporting Figure 3). Within the first 14 days, DTXL-μMESH returned a faster release rate as compared to PTXL-μMESH. Specifically, at day 14, 75.1 ± 4.3% of DTXL was released as opposed to only 41.4 ± 4.9% of PTXL, corresponding to cumulative drug amounts of ∼11 and 6 μg, respectively. In the following 4 months of observation, the release rates for both drug-μMESH configurations were similar and corresponded to an average of 0.8 ng/day following a quasi-linear release profile. Importantly, such daily release would return a local drug concentration of ∼1 nM in a 1 mL volume, which is still therapeutically effective as shown in the sequel. At the end of the release experiments, DTXL-μMESH released all of its drug content whereas about 40% of PTXL was still entrapped within the μMESH edges. Indeed, comparing DTXL-μMESH and PTXL-μMESH (Figure 2D), the amounts of drug released at 1 h, 14 days, and 90 days were statistically identical only for the first time point with ∼2.8 μg released in 1 h (*p* = 0.11). At the later time points, the amounts of released DTXL were about 3-fold higher than for PTXL.

The release of PTXL could be slower also because of the different solubilities in water of DTXL and PTXL, being ∼5 μg/mL for the first and only ∼0.5 μg/mL for the latter, and this can slow down the passage of PTXL from the polymer to the receptive phase (i.e., PBS).^27,28^ As such, DTXL would diffuse more rapidly than PTXL within the physiological solution, permeating the PLGA matrix of μMESH and, eventually, reaching the receptive media. Indeed, this behavior should be attributed to the specific interaction among the drug molecules, the PLGA matrix, and the surrounding physiological media. A moderate burst release during the first few days of operation, corresponding to about 20% of the loaded drug amount, is also observed. This should be mostly associated with the drug molecules residing closer to the PLGA/media interface. The large gradient in drug concentration between the PLGA (drug rich) phase and media (initially zero drug concentration) pushes DTXL and PTXL to rapidly diffuse out of the polymeric matrix. It is, however, important to recall here that, in cancer therapies, an initial faster release of cytotoxic molecules is indeed required to immediately act on the rapidly dividing malignant cells. Incidentally, this might also be the reason DTXL tends to be more cytotoxic than PTXL.

Note that the release studies were conducted for all the different drug input μMESH configurations, as documented in Supporting Figure 4. Interestingly, after analyzing the series of data for both molecules, it was observed that the release rates inversely correlated with the loaded drug amounts in the case of DTXL. In other words, the higher the initial DTXL input, the slower was the release rate with a difference of about 70% between the two extreme loading conditions, namely, 250 and 5000 μg of input DTXL. Conversely, for PTXL, the release rates were relatively independent of the initial drug input, returning a difference of only 30%. These data continue to highlight the importance of the interaction among therapeutic molecules, solvents, and materials in the design and realization of drug delivery systems. The documented differences between DTXL and PTXL should be again interpreted as referring to the different drug solubilities and the consequent formation of drug crystals. Supporting Figure 4 shows that, for low drug inputs (≤1500 μg), the release profiles associated with DTXL-μMESH and PTXL-μMESH were relatively similar, with slightly lower release rates for the latter than the former μMESH configuration. On the other hand, significant differences appeared for high drug inputs (≥2500 μg), for which the release rates associated with DTXL-μMESH were much lower than for PTXL-μMESH. Indeed, at higher input amounts, the occurrence of DTXL crystals, as opposed to molecular PTXL, would correlate with the slower release documented for DTXL-μMESH against PTXL-μMESH.^29^

### Pharmacological Characterizations of Nanomedicine-Loaded μMESH

**Figure 3:**
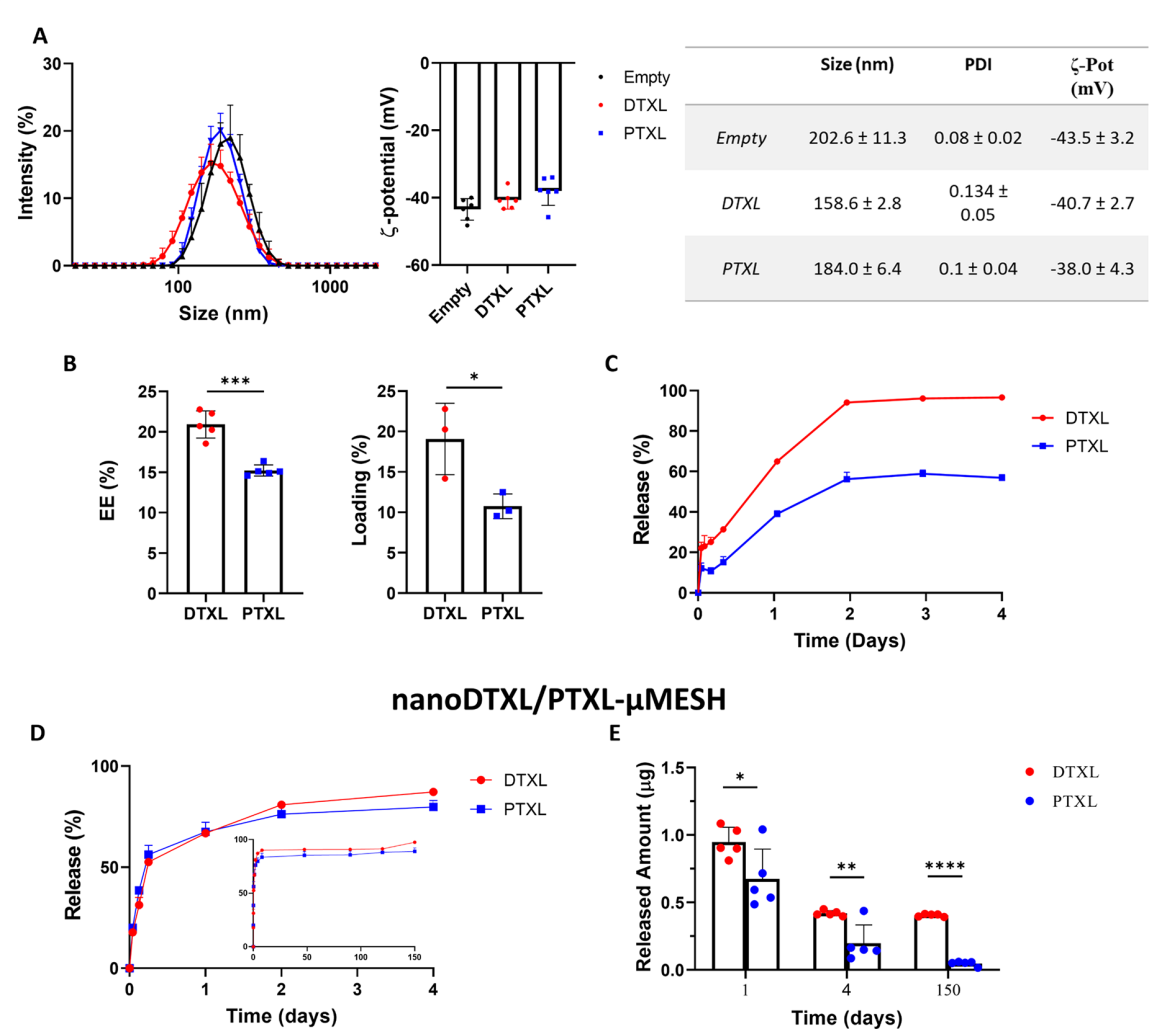
Pharmacological characterization of nanoDTXL and nanoPTXL loaded in μMESH. A. Size distribution (left) and surface ζ-potential (right) for nanoDTXL, nanoPTXL, and empty nanoparticles. The table summarizes the physicochemical properties of the three nanoparticle formulations. (B) Encapsulation efficiency (EE) and loading for nanoDTXL and nanoPTXL (***, *p* = 0.0001; *, *p* = 0.037). (C) Drug release profile from nanoDTXL and nanoPTXL over a 4 day observation period. (D) Drug release profile from μMESH over a 4 day observation period (the inset provides the release profile up to 150 days). (E) Amount of drug released from nanoDTXL-μMESH and nanoPTXL-μMESH at 1, 4, and 150 days (*, *p* = 0.039; **, *p* = 0.007; ****, *p* < 0.000001).

The release of the two anticancer drugs was also characterized in the case in which they were first formulated into nanoparticles (nanoDTXL and nanoPTXL) and, then, entrapped within the PVA microlayer of μMESH. The spherical nanoparticles presented a PLGA core externally stabilized by a lipid monolayer (Figure 1E,F) and were realized using the same parameters for nanoDTXL and nanoPTXL with a 3 mg drug input per preparation. In both formulations, the nanomedicines had a homogeneous distribution with similar hydrodynamic diameters, being 158.6 ± 2.8 nm (PDI = 0.13 ± 0.05) for nanoDTXL and 184.4 ± 6.4 nm (PDI = 0.1 ± 0.04) for nanoPTXL, and surface ζ-potentials of −40.7 ± 2.7 mV for nanoDTXL and −38 ± 4.3 mV for nanoPTXL (Figure 3A). Interestingly, both nanoDTXL and nanoPTXL were slightly smaller in size than the corresponding empty nanoparticles, which have a hydrodynamic diameter of 202.6 ± 11.3 nm (PDI = 0.08 ± 0.02) and surface ζ-potentials of −43.5 ± 3.2 mV. Indeed, this was observed previously and should be ascribed to the hydrophobic nature of the two drug molecules that contribute to realizing a more compact PLGA core. Considering drug encapsulation, DTXL again appeared to be more efficiently entrapped into the PLGA-based nanomedicines, returning an EE of 20.9 ± 1.7% as opposed to 15.2 ± 0.7% associated with PTXL (*p* = 0.0001; Figure 3B). A similar trend was also observed in the case of loading, returning statistically different values of 19.1 ± 4.4% and 10.8 ± 1.5% for DTXL and PTXL, respectively (*p* = 0.037; Figure 3B). Note that, in the preparation of these nanomedicines, DTXL and PTXL were dispersed in chloroform, and no formation of drug crystals was observed. Therefore, the higher drug encapsulation should be ascribed to the stronger affinity of DTXL for PLGA, as also proven by the smaller size of nanoDTXL compared to nanoPTXL. The release profiles from the nanoparticles for the two anticancer molecules were also assessed, showing a slower release for PTXL over DTXL (Figure 3C).

After a modest initial burst, nanoDTXL released its therapeutic content in about 4 days, whereas ∼40% of the drug was still entrapped in nanoPTXL at the same time. Finally, the release of DTXL and PTXL loaded into nanomedicines from μMESH was characterized for a 15 μg of drug total loading, corresponding to the dose used for the preclinical *in vivo* therapeutic experiments. Note that, consistent with the data of Figure 3B, a slightly lower amount of nanoDTXL (1.1 mg) had to be dispersed in the PVA microlayer as compared to nanoPTXL (1.3 mg) in order to achieve the same 15 μg of drug loading. This was further characterized as documented in Supporting Figure 5. For these μMESH loading configurations, a biphasic release behavior was anticipated whereby, at first, intact nanomedicines are released in the surrounding medium upon dissolution of the water-soluble PVA microlayer and, then, the anticancer drugs diffuse out of the nanomedicines. The resulting DTXL (red curve) and PTXL (blue curve) release profiles are shown in Figure 3D: a rapid release is observed within the first 6 h, which should be ascribed to the PVA microlayer dissolution, followed by a sustained release up to 4 days. Notably, over 60% of the payload was already available within the first 24 h. Nonetheless, drug release was documented over a much longer time scale, up to 150 days (Figure 3D,E). This should be associated with residual portions of the PVA microlayer, more intimately connected with the PLGA micronetwork, which would slowly dissolve and act as a longer-lasting depot for the nanomedicines (Supporting Figure 6). Indeed, a direct comparison between the released amounts at different time points, namely, 1, 4, and 150 days, documents a statistically significant difference between DTXL and PTXL which has to be ascribed to different molecular interactions arising between the payload and μMESH (Figure 3E). In particular, the amount released at 150 days is almost 7 times higher in the case of DTXL than PTXL.

Release experiments were also performed at pH 6.5, which is typically associated with the tumor microenvironment, for both μMESH carrying molecular and nanoDTXL and PTXL (Supporting Figure 7). Specifically, Supporting Figure 7A shows the release of DTXL (red lines) and PTXL (blue lines) from spherical polymeric nanoparticles encapsulated in the PVA microlayer. In line with numerous other reports, the acidic microenvironment (dashed lines) tends to slightly increase (∼5%) the release as compared to the physiological conditions (solid lines). More interesting is the behavior observed for the μMESH directly loaded with the drug molecules (Supporting Figure 7B). In this case, the release of the drug molecules from the PLGA micronetwork of μMESH was slowed rather than accelerated. Also, the reduction in release rates was larger for the less hydrophobic DTXL (red lines) as compared to PTXL (blue lines). This unexpected behavior should be ascribed to the p*K*_a_ (>10) of both drugs.^30,31^ This makes DTXL and PTXL less ionizable and therefore soluble in an aqueous solution at lower pH. Indeed, within the first days of observation, drug release is mostly governed by pure diffusion following three consecutive steps: permeation of the aqueous solution within the hydrophobic PLGA matrix; drug ionization and solubilization in the aqueous solution; drug diffusion from the PLGA matrix out in the surrounding medium. As such, a reduction in the water solubility of the drug molecules would also decrease their release rates. Furthermore, this is expected to affect DTXL more than PTXL, given the lower hydrophobicity of the former.

### Cytotoxicity Studies of μMESH on Glioblastoma Cell Cultures and Spheroids

**Figure 4:**
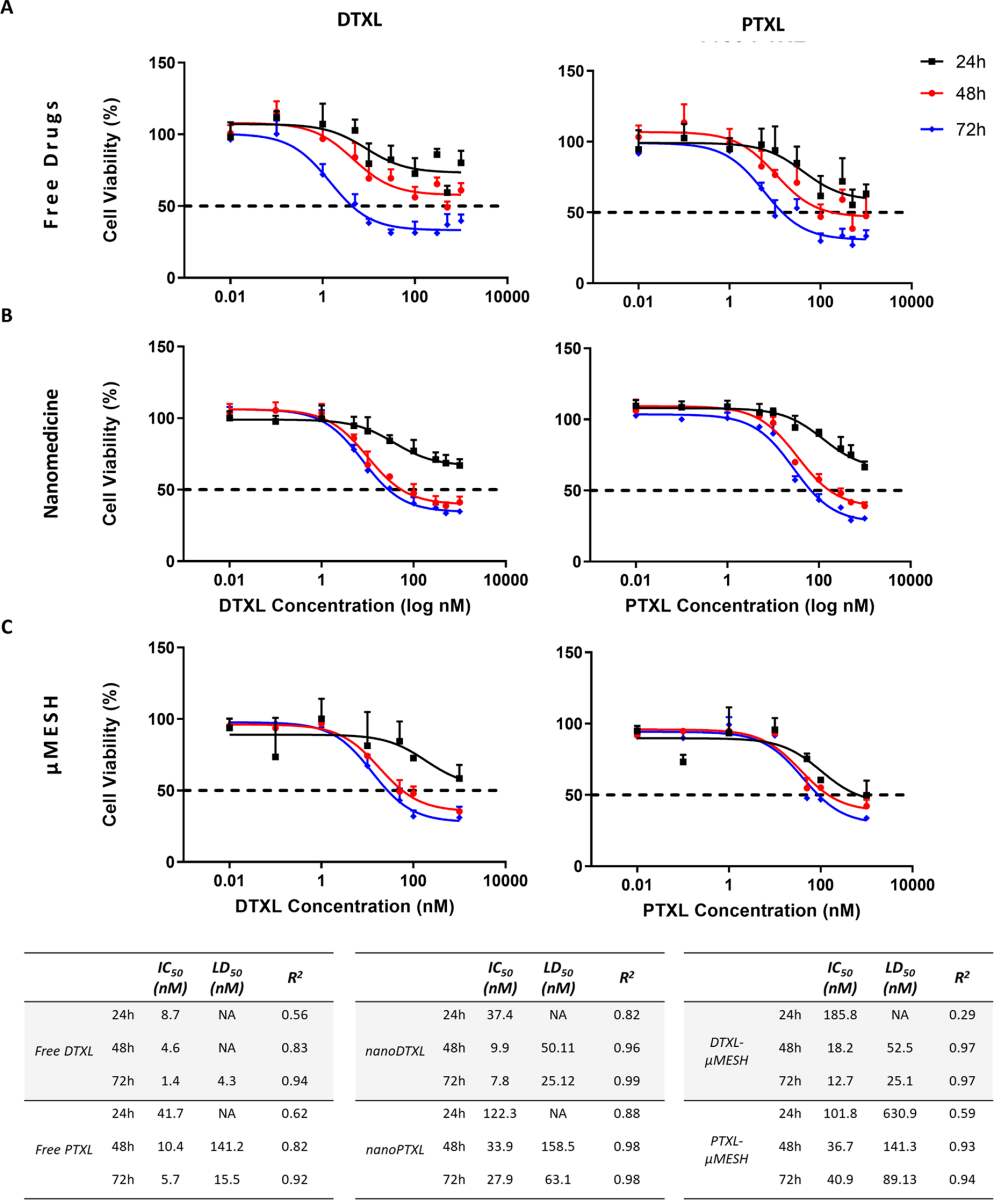
Viability of U-87MG cells (monolayer). 24, 48 and 72 h cell viability upon exposure to (A) free drugs; (B) nanoformulated drugs; (C) drug-loaded μMESH for various concentrations of the cytotoxic agents. The table collects the IC_50_, LD_50_, and *R*^2^ fitting values for all of the tested conditions.

First, monolayers of human glioblastoma cells (U-87 MG) were exposed to different concentrations of DTXL and PTXL for 24, 48, and 72 h (Figure 4). For these cytotoxicity studies, three different therapeutic groups were considered, namely, free molecules (Figure 4A); nanoparticle-loaded molecules (Figure 4B); μMESH (Figure 4C). As expected, at each given time point, the free drugs were more effective than the nanoformulated drugs, and the latter were more effective than the drug-loaded μMESH. This can be readily derived by comparing the IC_50_ and LD_50_ values across the different therapeutic groups and time points (see the table in Figure 4). Indeed, the IC_50_ and LD_50_ values of the free drug molecules at 24 h post-incubation are comparable with the 48 h time point of the nanomedicines and the 72 h time point of μMESH. From the table, it can also be inferred that DTXL is ∼3 times more potent (lower IC_50_ and LD_50_) than PTXL, on this specific cell line, for almost all the therapeutic groups and time points. As both taxanes have the same mechanism of action,^23^ these results reflect the fact that DTXL is known to be more effective than PTXL,^32^ as also confirmed by the data in Figure 4A. Notably, the LD_50_ values for DTXL-μMESH and PTXL-μMESH are comparable with those of nanoDTXL and nano-PTXL at 48 and 72 h. This would suggest that the rates of cell uptake and intracellular release for the nanoformulated drugs is similar to the release of the same free drugs from μMESH. As such, for a fixed total drug amount, μMESH and nanoformulated drug would be comparable.

**Figure 5:**
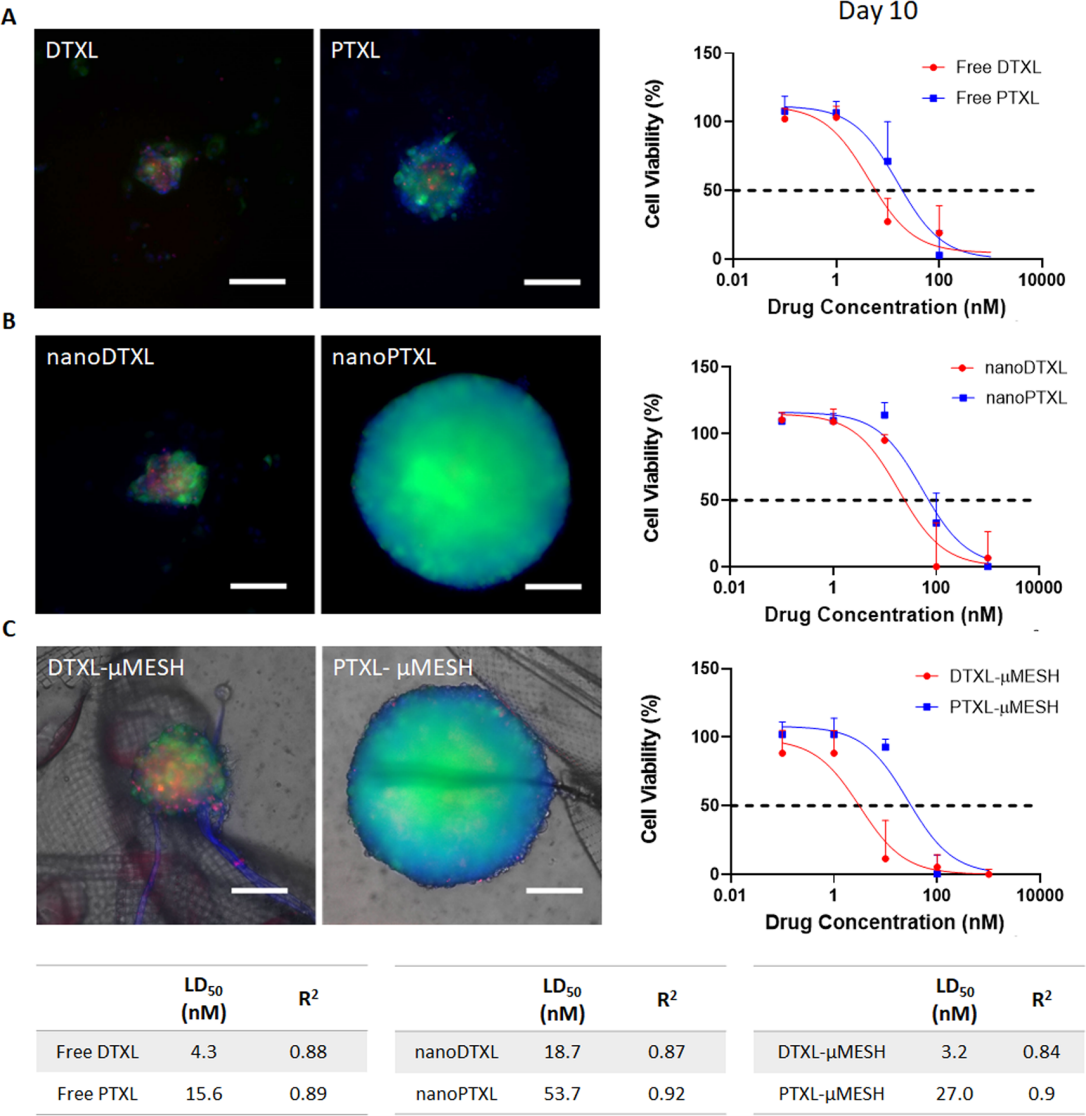
Viability of U-87MG cells (spheroids). Representative images (left) after 10 days of U-87MG tumor spheroids treated at 10 nM of (A) free drugs; (B) nanoformulated drugs; (C) drug-loaded μMESH and, in the right column, the corresponding cell viability curves, determined for various drug concentrations at day 10 post-treatment initiation (Scale bar: 200 μm). The table shows the LD_50_ and *R*^2^ fitting values for all of the tested conditions.

Similar experiments were performed on GBM spheroids to reproduce, in part, the biological complexity of the *in vivo* 3D cell network organization, presenting a necrotic core and subsets of cells with different levels of oxygenation and nutrient distribution as well as the inherent limitations in drug and nanoparticle diffusion. In these experiments, two parameters were monitored: the size of the tumor spheroid and the percentage of dead cells. Using the fluorescence signal associated with the U-87 MG GFP^+^, the equatorial diameter of the tumor spheroid could be readily measured over time for the different treatment modalities (Supporting Figures 8 and 9). As expected, higher drug concentrations corresponded to slower growth rates for all of the tested therapeutic groups. Notably, the minimum effective concentration, defined as the drug concentration required to return quasi-flat growth curves, varies for free, nano, and μMESH formulated drugs as well as for DTXL versus PTXL. In fact, when exposed to free drug molecules, spheroids exhibit a quasi-null growth rate already at 10 nM in the case of DTXL and for PTXL. However, when analyzing nano- and μMESH formulated drugs, the growth of the spheroids can only be halted at 10 nM for DTXL and ∼100 nM for PTXL. A similar response was also observed for the viability of the tumor cells within the spheroids, which was correlated to the percentage of dead cells at the end of the observation period (i.e., 10 days). Figure 5 and Supporting Figures 8 and 9 document the advantage of DTXL over PTXL in all the treatment groups. Importantly, in tridimensional assays, which more accurately recapitulate the complexity of the *in vivo* scenario as opposed to easily accessible cell monolayers, DTXL-μMESH was found to be as effective as free DTXL in inducing cell death. Also, in general, the μMESH configurations were more effective than the nanoformulated drugs possibly due to the lower penetration depth of the nanomedicines over the free molecules and the intimate interaction of μMESH with the tumor spheroid. Indeed, the authors have previously shown how μMESH would tend to entangle with and, at times, be pulled inside the growing malignant mass (Supporting Figure 10), thus facilitating drug distribution and penetration.^22^

### Preclinical Validation of μMESH in Orthotopic Murine Models of Glioblastoma

**Figure 6:**
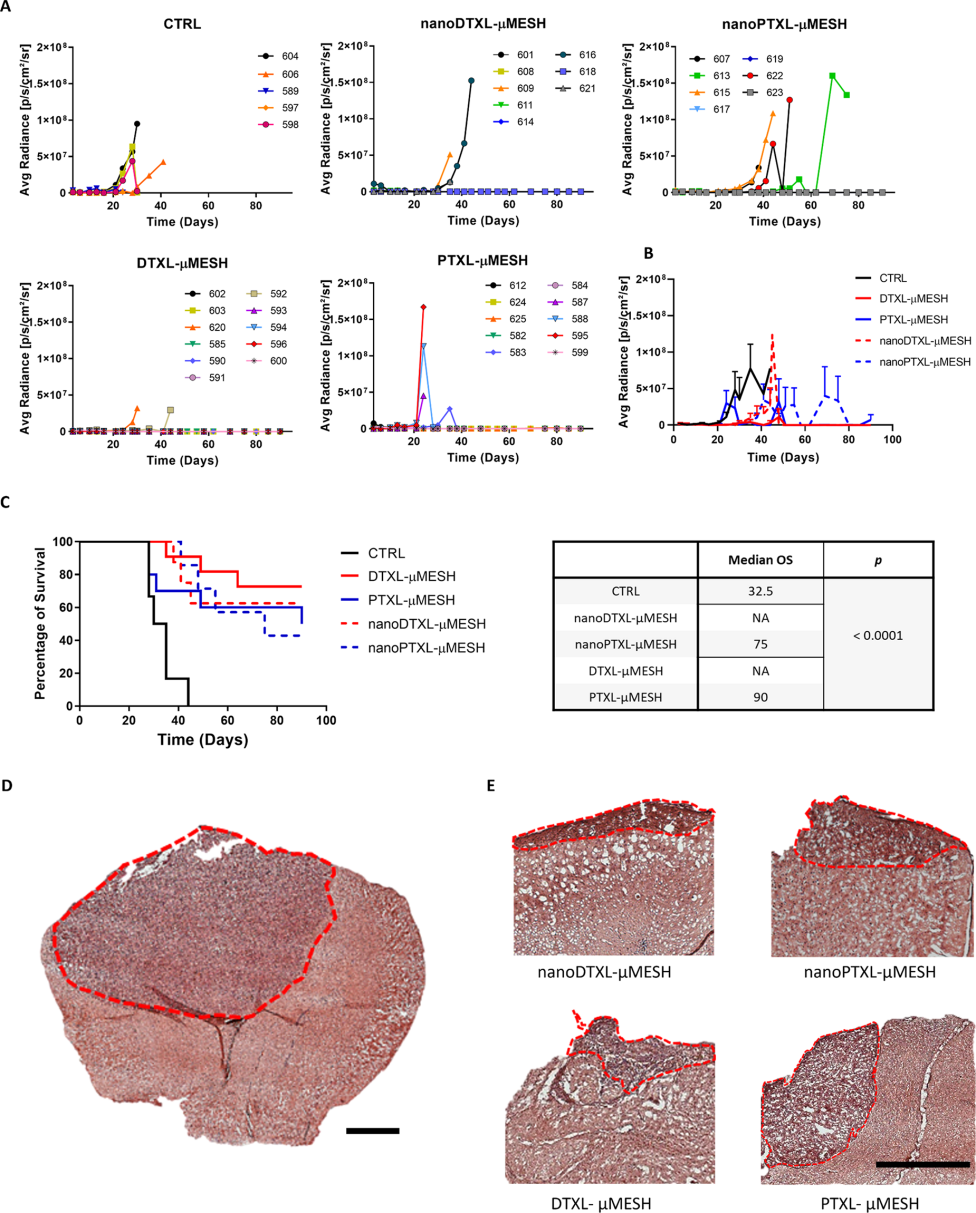
Preclinical therapeutic efficacy of μMESH on orthotopic models of glioblastoma. (A) Variation of the bioluminescence (BLI) signals over time for four therapeutic groups, including nanoDTXL-μMESH, nanoPTXL-μMESH, DTXL-μMESH, PTXL-μMESH, and the untreated control group (CTRL). (B) Average BLI signals for all five tested groups. (C) Kaplan–Meier survival curves for all five tested groups. The *p* column presents the two-sided log-rank test. (D) Representative brain tissue histological image at day 30 for the untreated control group (OS: 32.5 days) (scale bar: 1 mm). (E) Representative brain tissue histological images at day 90 for treated mice (scale bar: 1 mm).

The therapeutic efficacy of drug loaded μMESH (DTXL-μMESH and PTXL-μMESH) and nanomedicine loaded μMESH (nanoDTXL-μMESH and nanoPTXL-μMESH) was assessed in a rigorous orthotopic murine model of glioblastoma. The model was established by inoculating U-87 MG Luc^+^ cells in the brain of mice and waiting for 2 weeks before starting any treatment. This would allow the malignant mass to reach a level of development for which spontaneous regression would be unlikely. At the time of treatment initiation, the mice were randomly divided into groups for the different treatment regimes. It is here important to highlight that the authors have already demonstrated in a previous work on this same tumor model that the intravenous administration of free drugs and nanomedicine would not improve survival over the control group and, therefore, abiding to the spirit of reducing animal experiments, these additional control groups have not been included in the study.^22^ The growth of the tumor in each experimental group was monitored over time via bioluminescence imaging (BLI). Figure 6A reports the individual radiance values versus time for all 4 treatment groups and the control animals. Notably, the μMESH groups modulated the tumor growth over a longer period of time as opposed to the control group, whose mice had to be all sacrificed before day 40. This is summarized in the curves of Figure 6B that provides the average BLI values over time for the different 5 groups.

Perhaps more interesting are the survival data presented in Figure 6C. The median overall survival (OS) was 32.5 days for the untreated (CTRL) animals, in agreement with previous studies by the authors^22,33^ (Supporting Figure 11); 75 days for the nanoPTXL-μMESH; 90 days for the PTXL-μMESH. For the DTXL groups, the OS could not even be defined, as for both nanoDTXL-μMESH and DTXL-μMESH, over 50% of the mice survived longer than the observation period of 3 months. Specifically, 80% and 60% of the mice were still alive at day 90 in the DTXL-μMESH and nanoDTXL-μMESH groups, respectively. Despite the large advantage in survival over the untreated animals (*p* < 0.0001), no statistically significant difference was detected among the four therapeutic groups.

However, the trends depicted in Figure 6C would suggest that the DTXL groups (red lines) could be more effective than the PTXL groups (blue lines) and that small molecules distributed within the μMESH edges of the PLGA micronetwork (solid lines) could be more effective than μMESH carrying the nanoformulated drugs within the PVA microlayer (dashed lines). Again, these are only trends that could be fully verified by performing longer studies involving a larger number of animals. However, it is important here to highlight that the observed trends are in line with the cytotoxic LD_50_ data on tumor spheroids of Figure 5, showing a higher potency of free DTXL over free PTXL, of DTXL-μMESH over PTXL-μMESH, and of drug-loaded μMESH over nanomedicine-loaded μMESH. Also, it is important here to remark that DTXL and PTXL are released in a more sustained manner from DTXL/PTXL-μMESH (Figure 2C) as compared to nanoDTXL/nanoPTXL-μMESH (Figure 3D). Incidentally, these results again highlight how the proper release of therapeutic molecules could boost their native efficacy. Finally, Figure 6D shows the tumor burden in the control group at 30 days post-cell inoculation, clearly highlighting the aggressiveness and rapid growth of the malignant mass, which occupies almost 50% of the brain hemisphere. On the other hand, after 90 days, the μMESH-treated mice presented a much smaller malignant area, confirming the efficacy of the intervention (Figure 6E).

## Study Limitations

High grade gliomas are nearly uncurable with a 5-year survival of only 5%. The results presented in this work demonstrate that the proper reformulation and sustained delivery of conventional chemotherapeutic molecules, DTXL and PTXL, can dramatically extend the survival of mice bearing orthotopic glioblastomas. This is significant given the revived interest in treating high grade gliomas with poorly brain permeable and highly cytotoxic drugs, as documented by the recent approval of clinical studies where recurrent glioblastoma patients are treated with albumin-bound PTXL upon permeabilization of the blood–brain barrier via multiple, localized ultrasound sources.^34^ Despite all of this, the work has some limitations. First, brain tumors are obtained via intracranial inoculation of human U87-MG cells in immunocompromised mice. Recent analyses have shown that, over the years, the commercially available U87-MG cells have lost the genetic signature of the original patient-derived brain cancer cells.^35^ Nonetheless, these cells are still representative of human glioblastoma. Future studies should focus on syngeneic models, possibly implanting murine cancer cells such as CT-2A and GL261, in fully immunocompetent animals. Second, no statistical difference in overall survival was documented among the four different therapeutic groups, including nanoDTXL-μMESH, DTXL-μMESH, nanoPTXL-μMESH, and PTXL-μMESH, after observing 8 mice per group for up to 90 days. Although clear trends can be observed with the DTXL-μMESH leading the group, future studies should consider longer observation periods and a larger cohort of animals to unequivocally identify the most effective therapeutic strategy.

## Conclusion

A deformable and biodegradable implant, μMESH, was demonstrated for the sustained release of different therapeutic agents from a peritumoral position toward unresected masses of high-grade brain tumors. The dual compartmentalized μMESH was carrying chemotherapeutic molecules docetaxel (DTXL) or paclitaxel (PTXL), dispersed within the PLGA micronetwork, or nanomedicines loaded with DTXL and PTXL (nanoDTXL and nanoPTXL) encapsulated within the PVA microlayer. The four resulting μMESH configurations were extensively characterized for their morphological and pharmacological properties as well as therapeutic efficacy on orthotopic models of glioblastoma. Results documented the continuous delivery of the potent chemotherapeutic agents for at least 150 days. However, while about 80% of nanoDTXL and nanoPTXL was released within the first 4 days of operation, it took 14 days to release 75% and 45% of molecular DTXL and PTXL, respectively. Notably, the unique compartmentalized architecture of μMESH, including a regular micronetwork of PLGA edges intercalated over a PVA microlayer, offers the rare opportunity to realize deformable implants with different loading and release conditions for the same molecules in order to identify the configuration with the highest chemotherapeutic potential. Indeed, all four μMESH configurations were tested on tridimensional spheroids of U87-MG tumor cells, demonstrating that DTXL-μMESH required the lowest drug dose to inhibit disease progression, followed by nanoDTXL-μMESH, PTXL-μMESH, and nanoPTXL-μMESH. Similar trends were also documented *in vivo* where μMESH was deposited over the surface of unresected masses of U87-MG cells inoculated intracranially and left to grow for 15 days before treatment. nanoPTXL-μMESH and PTXL-μMESH returned an overall survival of 75 and 90 days, respectively, which was about 2.5 to 3 times higher than that for the untreated mice (32.5 days). On the other hand, 60% and 80% of the nanoDTXL-μMESH and DTXL-μMESH treated mice, respectively, were still alive at the end of the 90 days of observation. All together, these results would suggest that, in addition to identifying more selective inhibitors, the sustained delivery for several weeks from a peritumoral position of small doses of potent chemotherapeutic molecules properly packaged in a conformable polymeric implant could halt the progression of aggressive brain tumors.

## Materials and Methods

### Materials

Poly(d,l-lactic-*co*-glycolic) acid (PLGA), poly(vinyl alcohol) (PVA), acetonitrile, analytical grade dimethyl sulfoxide (DMSO), chloroform, sucrose, and Gill’s No. 2 Hematoxylin solution Eosin Y solution were purchased from Merck KGaA (Darmstadt, DE). Poly(dimethylsiloxane) (PDMS) silgard 184 was obtained from Dow Corning (Germany). 1,2-Dipalmitoyl-*sn*-glycero-3-phosphocholine (DPPC) and 1,2-distearoyl-*sn*-glycero-3-phosphoethanol-amine-*N*-[carboxy(polyethylene glycol)-2000] (DSPE-PEG) were purchased from Avanti Polar Lipids (Alabaster, AL, USA). Docetaxel and paclitaxel were purchased from Alfa Aesar (Haverhill, Massachusetts, USA).

### μMESH Fabrication and Characterization

μMESH is prepared following a soft lithographic method previously described by the authors.^22^ Briefly, a silicon master template reproducing the main geometric features of the final μMESH structure was realized by direct laser writing (DLW). Specifically, 3 μm wide ridges delineate a regular matrix of 20 × 20 μm square pillars. The depth of the channels, corresponding to the height of the pillars, is 5 μm. Note that all of these geometrical parameters can be readily changed by reprogramming the DLW step. The silicon master template was replicated into a PDMS layer by pouring the polymeric base with a curing agent (10:1 ratio) on top of the patterned silicon surface and letting it dry at 60 °C for 4 h. Then, the PDMS template was replicated into a PVA microlayer upon evaporating for 2 h at 60 °C; the aqueous content of the polymeric solution was poured over the PDMS template. The resulting PVA microlayer, which precisely reproduces the geometrical pattern of pillars and ridges as in the original silicon template, was peeled off from the PDMS. The network of 3 × 5 μm ridges realized on the surface of the PVA microlayer was carefully filled with a polymeric paste obtained by dissolving 5 mg of PLGA in acetonitrile (63 μL). The resulting 27.5 × 27.5 mm μMESH, comprising the PVA microlayer and the PLGA micronetwork, was cut into 30 different pieces of 5 × 5 mm in size. All the *in vitro* and *in vivo* experiments were conducted with the 5 × 5 mm μMESH.

μMESH carrying docetaxel (DTXL) or paclitaxel (PTXL) was prepared by adding known amounts of the two drugs in the original PLGA paste before the ridge filling step. μMESH carrying nanoparticles loaded with docetaxel (nanoDTXL) or paclitaxel (nanoPTXL) was prepared by adding known amounts of the nanomedicines in the original PVA solution before its deposition over the intermediate PDMS template. Then, the resulting PVA solution enriched with nanoparticles was dried by following the same procedure for the preparation of the PVA microlayer.

The fine geometrical structure of μMESH was characterized via scanning electron microscopy (SEM) by sputtering 15 nm of gold before imaging each sample at 15 keV using an analytical low-vacuum SEM instrument (JSM-6490, JEOL). For the pharmacological characterizations, the loading of DTXL or PTXL into the edges of the PLGA micronetwork and the related release over time off the μMESH were analyzed by HPLC (1260 Infinity, Agilent Technology, U.S.A.) by acquiring the absorbance at 230 nm. Specifically, drug loading was determined by dissolving 5 × 5 mm DTXL (or PTXL) carrying μMESH (*n* ≥ 5) in a acetonitrile:water solution (1:1 ratio), sonicating the samples to accelerate polymer dissolution, and analyzing the resulting solution after centrifugation at 18,000*g* for 5 min. The cumulative amount of drug released was evaluated by placing 5 × 5 mm DTXL (or PTXL) carrying μMESH (*n* ≥ 4) in a small tube with 1 mL of PBS at 37 °C, simulating the volume of the body fluids to which the system would be exposed upon deposition within the brain. At predetermined time points, namely, 0, 1, 3, and 6 h and 1, 2, 4, 7, 14, 28, 34, 60, 90, 120, 150, and 180 days, samples were centrifuged at 18,000*g* for 3 min to pellet down the residual μMESH. Then, the supernatant was collected, diluted with acetonitrile (in a 1:1 ratio), and analyzed by HPLC as detailed above.

### Nanoparticle Fabrication and Characterization

Spherical polymeric nanoparticles were prepared by a sonication–evaporation method, previously optimized by the authors.^24,25^ In summary, 10 mg of PLGA, 0.9 mg of DPPC, and 3 mg of the desired drug, DTXL or PTXL, were dissolved in 800 μL of chloroform, acting as the organic phase. In the aqueous phase, constituted by a 4% ethanol solution, 1.1 mg of DSPE-PEG was dispersed. The organic phase was added in a dropwise manner to the aqueous phase under ultrasonication and then placed in a reduced pressure environment. After the evaporation of the organic solvent (3 h), the solution with nanoparticles was centrifuged first at 250*g* for 2 min to remove any debris and then washed three times at 15,000*g* for 15 min discarding the nonencapsulated drug.

The nanoparticle size distribution and surface ζ-potential were measured via dynamic light scattering (Nano ZS, Malvern, UK) by analyzing 1 mL of milli-Q water in which 20 μL of particle solution was dispersed (*n* = 6). A Folded Capillary Zeta Cell (Malvern, UK) was used for the surface ζ-potential measurements (*n* = 6). The nanoparticle geometry was also confirmed by SEM imaging, following the same step described above for μMESH.

For the pharmacological characterization, both the encapsulation and loading efficiency were determined, with the first (EE) being the percentage ratio of drug mass entrapped in the nanoparticles over the amount of input drug and the second (loading) being the percentage ratio of drug amount loaded in the nanoparticles over the total mass of the formulation (drug, polymers, and lipids). For these analyses, DTXL (or PTXL)-loaded nanoparticles (*n* = 5 for EE and *n* = 3 for loading) were lyophilized, dissolved in a acetonitrile:water (1:1) solution to break the particles in their individual components, and then analyzed by HPLC (1260 Infinity, Agilent Technology, U.S.A.). For the release studies, the same amount of nanoparticles (*n* = 3) was dispersed in PBS and placed in Slide-A-Lyzer MINI dialysis microtubes with a molecular cutoff of 10 kDa (Thermo Scientific) and exposed to 4 L of PBS, as the receptive phase. At each time point, namely, 1, 2, 4, and 8 h, and 1, 2, 3, and 4 days, the content of each cup was independently collected, centrifuged at 15,000*g* for 15 min to pellet the nanoparticles, which were then disrupted in acetonitrile:water (1:1) solution, and analyzed by HPLC. The release is represented as a cumulative percentage, obtained by adding at each time point the percentage amount of the previous one and setting the total initial amount to 100%.

Finally, nanoDTXL (or nanoPTXL) was loaded into μMESH by dispersing it in the PVA solution prior to its application over the PDMS template. Then, the resulting PVA solution enriched with nanoparticles (nanoDTXL- or nanoPTXL-μMESH) was dried by following the same procedure for the preparation of the PVA microlayer. For the release study of nanoloaded μMESH, 5 × 5 mm μMESH (*n* = 5) was placed in a 1 mL tube at 37 °C. At predetermined time points, namely, 0, 1, 3, and 6 h and 1, 2, 4, 7, 14, 28, 34, 60, 90, 120, and 150 days, samples were centrifuged at 18,000*g* for 3 min to pellet down the residual μMESH. The supernatant, which contains both drug-loaded nanoparticles and free drug molecules already released from the nanoparticles, was collected, diluted with acetonitrile (in a 1:1 ratio), and analyzed by HPLC as detailed above.

### Cytotoxicity Experiments on Glioblastoma Cells

The cytotoxic potential of nanoDTXL (or nanoPTXL) and μMESH was evaluated and compared to the free drug molecules on tumor cell monolayers (2D condition) and spheroids (3D condition). For the 2D testing, 5 × 10^4^ U-87 MG GFP^+^ cells were seeded in 24 well plates and, on the day after, exposed to different concentrations of the therapeutic agent of interest (free molecules, nano, μMESH) (*n* = 5). At each predetermined time point, namely, 24, 48, and 72 h, the cell culture medium was removed from each well; the cells were washed with fresh PBS, and an MTT assay (Sigma-Aldrich) was performed according to the manufacturer’s instructions. The percentage of viable cells was quantified as the ratio between the treated and untreated (control) groups, which was set to 100%. The obtained data were then fitted to generate survival curves from which IC_50_ and LD_50_ values were extracted. Conventionally, IC_50_ defines the concentration at which 50% of drug efficacy is achieved, while LD_50_ indicates the drug concentration at which 50% of the cells are dead. For the 3D testing, 500 U-87 MG GFP^+^ cells were used to generate individual spheroids, seeding them in Cell Carrier Spheroid ULA 96-well Microplates (PerkinElmer). After 1 day, spheroids were transferred to a 24-well plate and treated with different concentrations (0.1, 1, 10, 100, and 1000 nM) of the therapeutic agent of interest (*n* = 4). The intrinsic green fluorescence of the U-87 MG GFP^+^ cells was used to quantify the diameter of the tumor spheroid over time and define its growth rate as the percentage increase in size as compared to time 0. In addition, a ReadyProbes cell viability imaging kit (Molecular Probes) was used to quantify the percentage of live and dead cells within the spheroid. Propidium iodide (PI) in the kit stains specifically for the dead cells. Therefore, following standard procedures, survival curves were generated by measuring the fluorescence intensities associated with PI and assuming 100% cell viability for the untreated control group that has the lowest PI fluorescence and 0% cell viability for the treated group with the highest PI fluorescence. Therefore, in Figure 5, all the curves would eventually range between 100% and 0% because of the normalization process per se. Note that, even for the control group, the PI fluorescence is not exactly zero as some death cells are expected to be localized in the core of the spheroid where the amount of nutrients and oxygen is lower. Differently, in Figure 4, following standard procedures, all the viability data for the monolayer culture were normalized with respect to the control (untreated) group only and set conventionally to 100% viability. As such, no 0% viability values were preset in the normalization process.

### Preclinical Therapeutic Experiments on Orthotopic Murine Models of Glioblastoma

Orthotopic mouse models of glioblastoma were realized using luciferase-positive U-87 MG Luc^+^ cells to monitor tumor growth longitudinally via bioluminescence imaging (BLI) analyses. Specifically, 5 × 10^5^ U-87 MG Luc^+^ cells were suspended in 3 μL of cold PBS and injected, with a rate of 0.3 μL/min, using a 10 μL sterile Hamilton syringe (26-gauge needle) attached to a stereotaxic frame, into the right hemisphere of the mouse brain (1.5 mm posterior to the bregma, 1.4 mm lateral to the midline) at a depth of 1 mm from the skull. Wounds were then closed with sterile clips, and the animals were carefully monitored until they recovered from anesthesia. Tumor growth was assessed every 2 days using an IVIS Spectrum (PerkinElmer) after intraperitoneal injection of a luciferin solution (200 μL at 150 mg/kg). The BLI data were processed with the Living Image 4.5.5 software (PerkinElmer).

After approximately 2 weeks, which is the time required to have a stably growing mass that would not self-resolve, corresponding to a BLI signal of about 10^6^ p/s/cm^2^/sr, mice were randomly divided into 5 experimental groups, namely, μMESH carrying free DTXL molecules (DTXL-μMESH), free PTXL molecules (PTXL-μMESH), nanoDTXL particles (nanoDTXL-μMESH), and nanoPTXL particles (nanoPTXL-μMESH) and the untreated (CTRL) group. For all the therapeutic groups, DTXL and PTXL were administered at 0.75 mg/kg in a single application without removing the malignant mass.

At the end of the experiment (i.e., 90 days) or at the onset of neurological or systemic deficits, mice were euthanized; brains were collected and processed for histological analyses. After dissection, all the specimens were transferred in fixative solution (4% paraformaldehyde in PBS, Santa Cruz Biotechnology, Heidelberg, DE) for 24 h at 4 °C. Then, samples were washed once with PBS and prepared for cryosectioning by two incubation steps in 30% sucrose in PBS, each step for at least 24 h (until samples sink in the solution) at 4 °C. At the end of this process, samples were frozen with vapors of liquid nitrogen and transferred at −80 °C. Before sectioning, each sample was embedded in Surgipath FSC 22 Frozen Section Compound (Leica Microsystems GmbH, Wetzlar, DE). Serial coronal sections (12 μm thick) of tumor area were cut and processed for the Haematoxylin-Eosin (H&E) staining (Gill’s No. 2 Hematoxylin solution for 6 min, counterstained with Eosin Y solution for 5 s, then dehydrated in ethanol, cleared with xylene, and mounted in Permount Mounting Medium from ThermoFisher Scientific, Waltham, MA, US). Images were acquired at 4× magnification by an Olympus BX-51 upright microscope with a Optronic Microfire A/R camera, driven by a stage controller and Neurolucida software (MBF Bioscience).

### Statistical Analyses

The comparison between two groups was performed by an F-test to exclude the presence of significant variances between the two followed by a two-tailed *t* test. Multiple comparisons (i.e., 3 or more groups) were performed using the Brown–Forsythe test for the equality of variance among the groups, followed by a one-way analysis of variance test. The Tukey posthoc test was used to discriminate differences in two-pair comparisons. Survival curves were plotted by using the Kaplan–Meier method. The two-sided log-rank test was used to assess the statistical significance. All of the tests and graphs were realized using GraphPad Prism. Differences were considered statistically significant when returning a *p* value lower than 0.05.

## Supporting Information Available

Supplementary figures and graphs related to loading and release characterization of DTXL and PTXL in all discussed configurations, drug physical state analyses, cytostatic effect and interaction of μMESH with tumor spheroids, and *in vivo* data comparison (PDF)

## Notes

All animal experiments were performed according to the guidelines established by the European Communities Council Directive (Directive 2010/63/EU of 22 September 2010) and approved by the National Council on Animal Care of the Italian Ministry of Health. All efforts were made to minimize the number of animals required to produce statistically relevant results, according to the “3Rs” (replacement, reduction and refinement) concept, and animal suffering.
