# Supplementary material for: μMESH-Enabled Sustained Delivery of Molecular and Nanoformulated Drugs for Glioblastoma Treatment
Source: ACS Nano. 2023 Jun 28;17(15):14572–85. doi: 10.1021/acsnano.3c01574 (PMC10416560; doi:10.1021/acsnano.3c01574)
Supplement: Supplementary file 1 — nn3c01574_si_001.pdf [file nn3c01574_si_001.pdf]

Supporting Information

μMESH-ENABLED SUSTAINED DELIVERY OF MOLECULAR AND NANOFORMULATED DRUGS FOR GLIOBLASTOMA TREATMENT

Daniele Di Mascolo[1,2], Irene Guerriero[1,3], Cristiano Pesce[1,4], Raffaele Spanò[1], Anna Lisa Palange[1], Paolo Decuzzi[1♣]

[1] Laboratory of Nanotechnology for Precision Medicine, Fondazione Istituto Italiano di Tecnologia, Genoa, Italy

[2] Department of Electrical and Information Engineering, Politecnico di Bari, Italy

[3] Department of Bioengineering and Robotics, Università di Genova, Italy

[4] Department of Pharmaceutical and Pharmacological Sciences, University of Padua, Padova, Italy

♣ Corresponding author: Paolo Decuzzi, PhD – paolo.decuzzi@iit.it

Supporting Results and Figures

Loading of docetaxel (DTXL) and paclitaxel (PTXL) in µMESH. The correlation between input and loaded drug amounts (**Supplementary Figure 1**) and the spatial distribution of the loaded drug molecules (**Supplementary Figure 2**) are considered below.

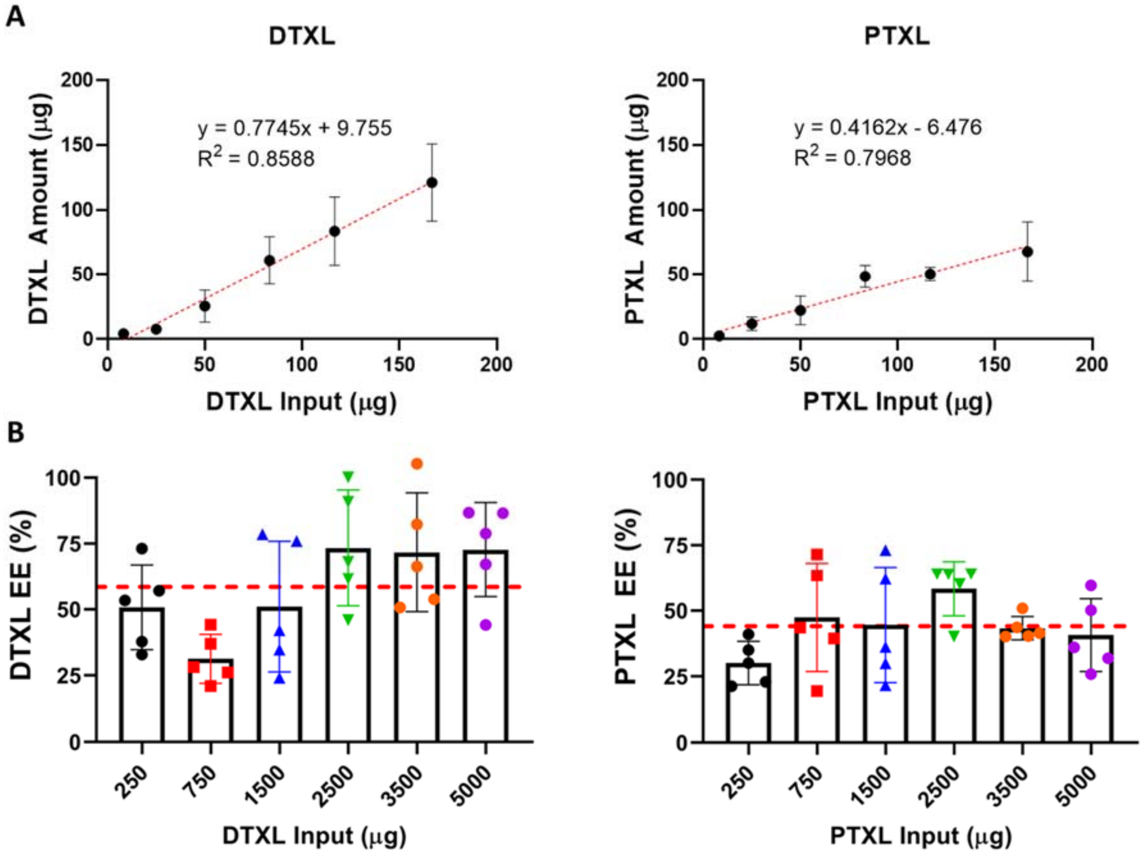

Supporting Figure 1. DTXL and PTXL loading in µMESH. A. Correlation between input and loaded DTXL (**left**) and PTXL (**right**) amounts in 5×5 mm pieces of µMESH. **B.** Encapsulation efficiency (EE) for all the tested µMESH configurations (different drug input amounts in 27.5×27.5 mm pieces of µMESH). Red dashed lines represent the average EE values for DTXL and PTXL.

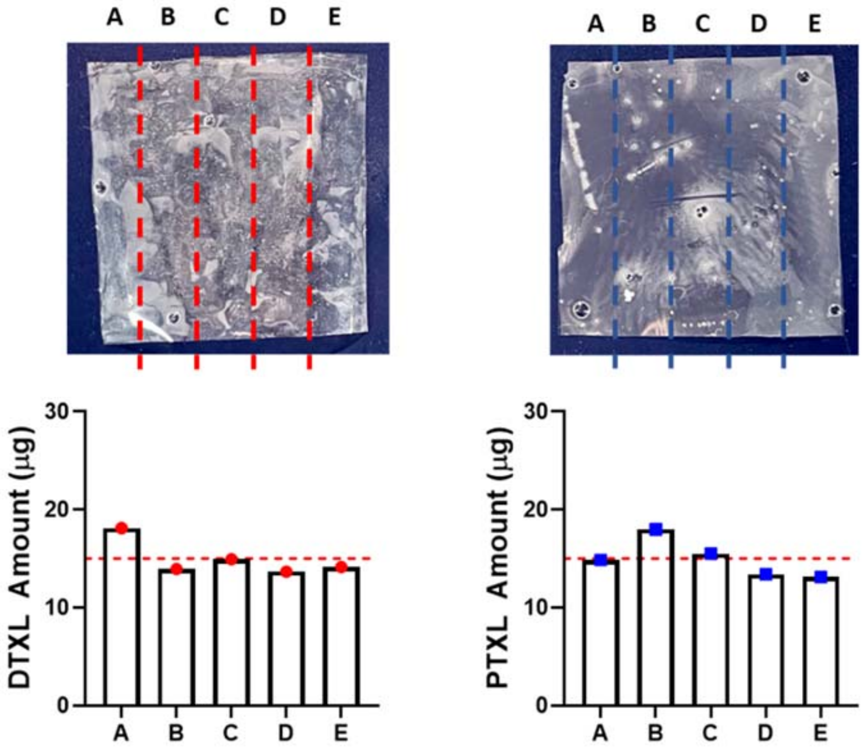

Supporting Figure 2. Spatial drug distribution in a full-sized 27.5×27.5 mm µMESH. The full sized µMESH was cut in 5 parallel rectangular stripes (**dashed lines, top**), from which 5×5 mm pieces of µMESH were further isolated and analyzed for drug content via liquid chromatography, for both DTXL (**left**) and PTXL (**right**). The bottom graphs demonstrate the uniform spatial distribution of drug molecules within the PLGA micronetwork.

Physical state of DTXL and PTXL in different solvents. The solubility of DTXL and PTXL in acetonitrile was assessed considering concentrations of 50 mg/ml, which are identical to those used for the µMESH fabrication. After 2 hours, an aliquot of each drug solution was taken and centrifuged at 15,000 *g* for 5 minutes to remove any precipitate. Then, the supernatant was analyzed via liquid chromatography to measure the drug concentration in the solution. This concentration corresponded to the maximum solubility of the drug in acetonitrile, returning values of 6.4 ± 0.9 and 46.13 ± 1.9 mg/ml for DTXL and PTXL, respectively (**Supporting Figure 3A)**. In the 50 mg/ml solutions, the formation of drug crystals was readily appreciated only in the case of DTXL as opposed to PTXL, as opposed to the drugs behavior when dissolved in chloroform (**Supporting Figure 3B)**.

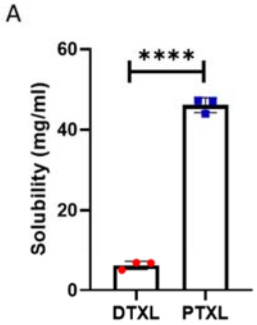

	250 µg	750 µg	900 µg	1000 µg	1500 µg	2500 µg	3500 µg	5000 µg
Final drug concentration in the spreaded paste (mg/ml)	3.96	11.9	14.3	15.9	23.81	25	35	44.4

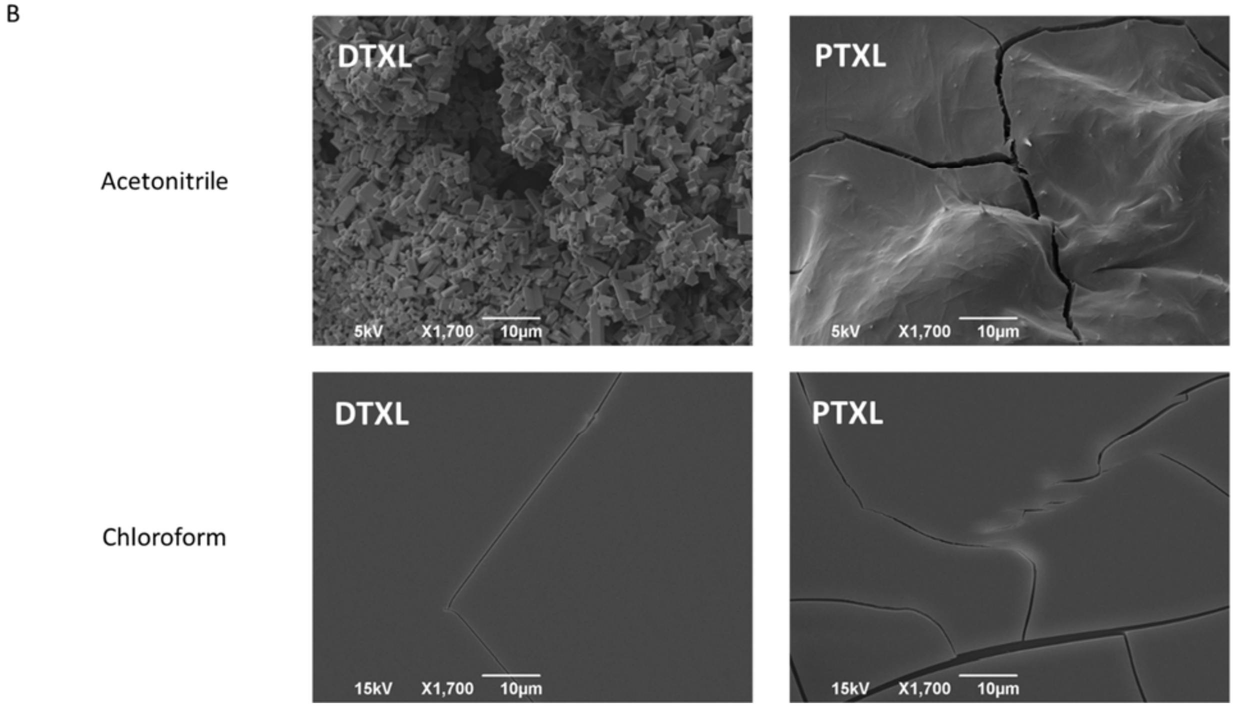

Supporting Figure 3. Physical state of DTXL and PTXL in different solvents. A. DTXL and PTXL solubility in acetonitrile (p = 0.000005). The table on the right collects the drug concentrations used to realize the µMESH for the different input values. **B.** In acetonitrile, at the concentration used for the µMESH fabrication (50 mg/ml), DTXL forms individual crystal-like structures (**left**), whereas PTXL forms a continuous layer (**right**), which is indicative of an amorphous state. In chloroform, at the concentration used for the nanomedicine fabrication (10 mg/ml), DTXL (**left**) and PTXL (**right**) both form a continuous layer, which is indicative of an amorphous state. No crystal formation is observed in chloroform (**lower row**), confirming the higher solubility of both drugs in this solvent.

Release of docetaxel (DTXL) and paclitaxel (PTXL) from μMESH.

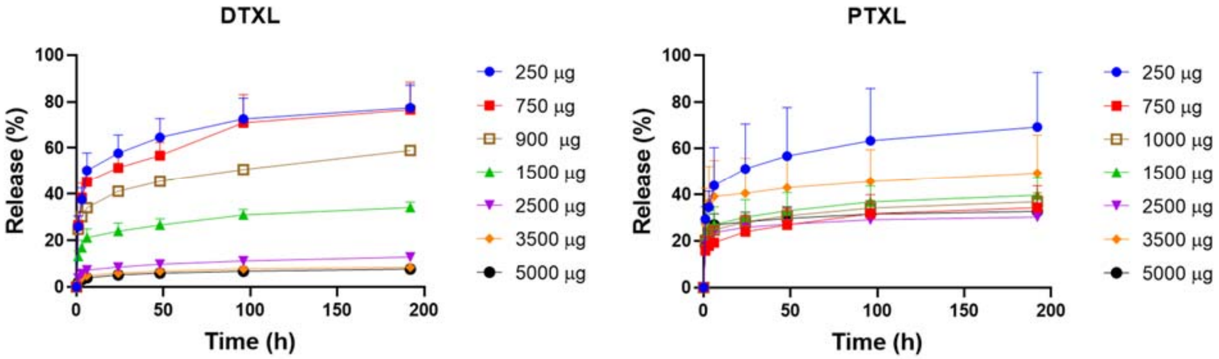

Supporting Figure 4. Drug release profiles from DTXL- and PTXL-μMESH for different loading conditions. (**left**) DTXL release rates decreasing with increasing initial drug loading input; (**right**) PTXL release rates being only moderately affected by initial drug loading input.

Loading and Release of nanoDTXL/nanoPTXL from μMESH.

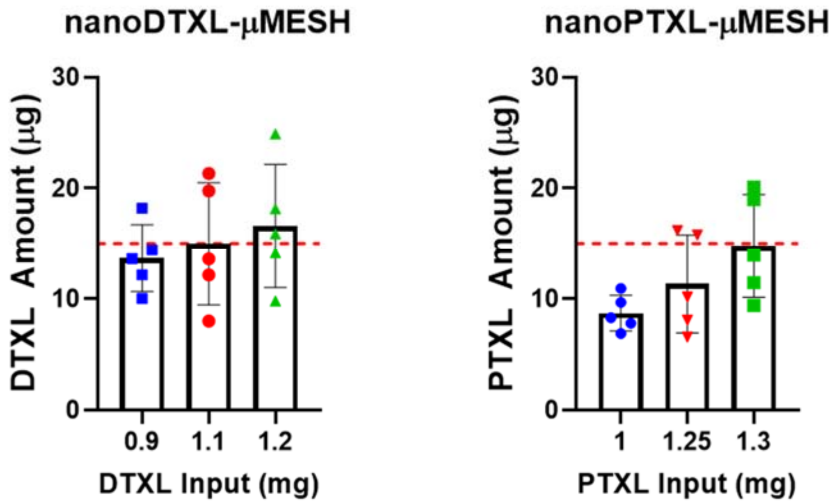

Supporting Figure 5. nanoDTXL/nanoPTXL-μMESH loading characterization. Amounts needed for nanoDTXL (**left**) and nanoPTXL (**right**) to fabricate μMESH with the same DTXL and PTXL amounts (i.e., 15 μg).

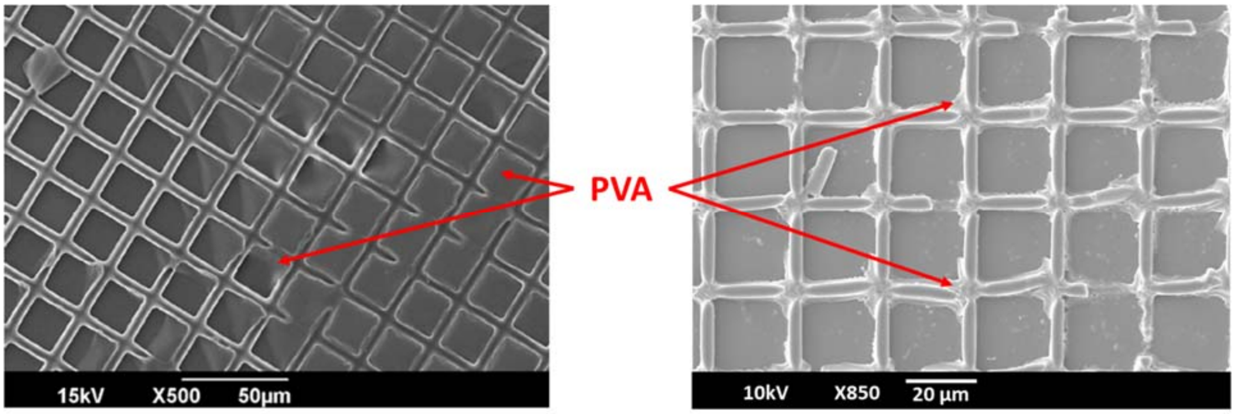

Supporting Figure 6. Dissolution of the PVA microlayer. Upon exposure to an aqueous solution (i.e., PBS), the PVA microlayer starts to dissolve. Scanning electron microscopy images of µMESH showing the progressive dissolution of the PVA microlayer upon exposure to PBS for 2 hours (**left**) and 2 weeks (**right**). Even after 2 weeks, residual amounts of PVA can be spotted attached at the corners of the PLGA micronetwork (**red arrows**). These are responsible for the sustained release of DTXL and PTXL for several weeks.

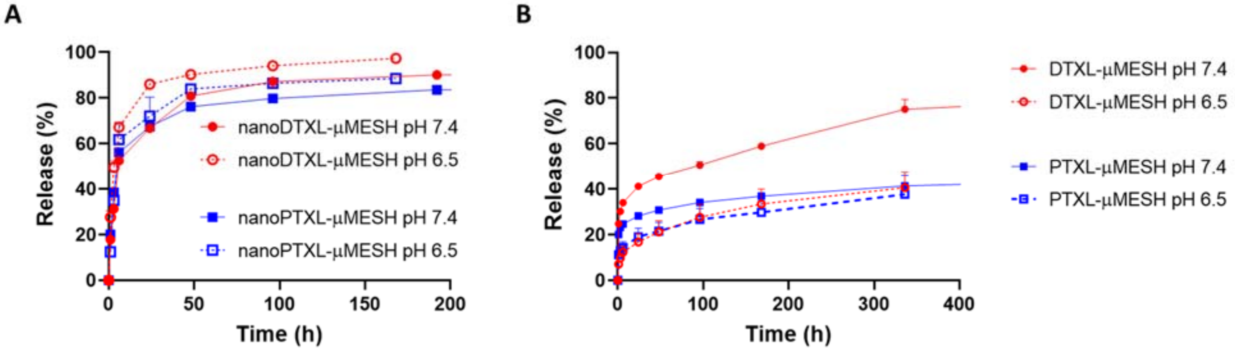

Supporting Figure 7. **pH dependent drug release profiles from µMESH**. **A.** Drug release from µMESH carrying nano-DTXL (red lines) and nano-PTXL (blue lines) into the PVA microlayer and exposed to physiological (solid lines; pH = 7.4) and slightly acidic (dashed lines; pH = 6.5) conditions; **B**. Drug release from µMESH carrying molecular DTXL (red lines) and PTXL (blue lines) loaded into the PLGA micronetwork and exposed to physiological (solid lines; pH = 7.4) and slightly acidic (dashed lines; pH = 6.5) conditions.

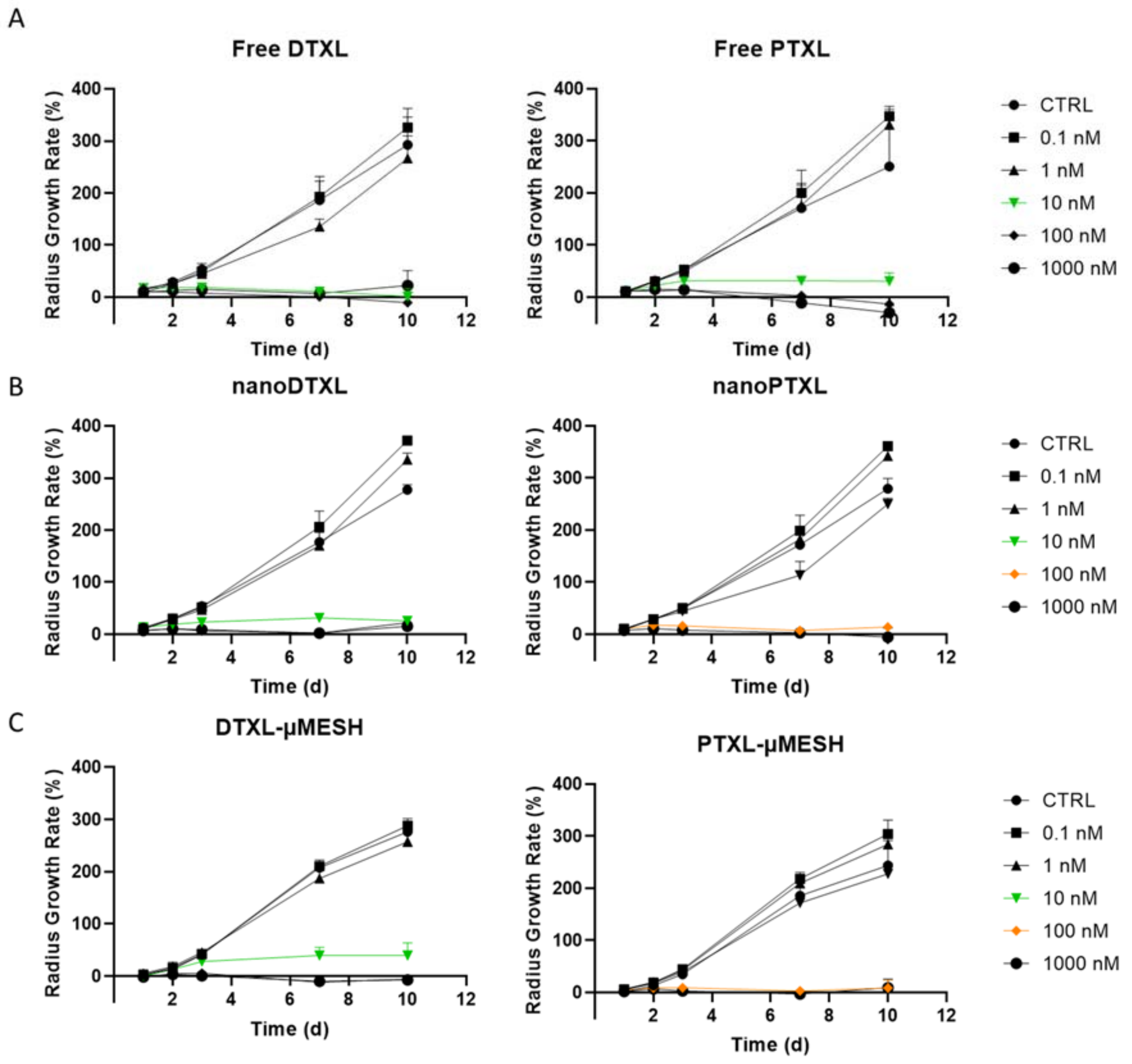

	Free DTXL	Free PTXL	nanoDTXL	nanoPTXL	DTXL-μMESH	PTXL-μMESH
CTRL	31.61	27.2	29.51	29.76	32.92	29.14
0.1 nM	35.4	37.2	38.83	37.66	33.98	35.41
1 nM	26.73	34.72	33.71	34.99	29.71	33.16
10 nM	-1.88	1.56	1.496	23.31	4.01	26.51
100 nM	-2.25	-2.68	1.045	-0.38	-1.31	-0.02
1000 nM	0.78	-5.01	0.35	-1.57	-1.16	0.2

Supporting Figure 7. Cytostatic effect on U-87MG spheroids – growth curves. Variation of the U87MG spheroid radius over time following exposure to free drugs (**A**), nanomedicines (**B**), and drug-loaded μMESH (**C**). The table at the bottom collects the values of the slope at time 0 for

all the tested conditions. A slope ≤ 5 (red values in the table) was associated with the minimum effective concentration (green and orange curves).

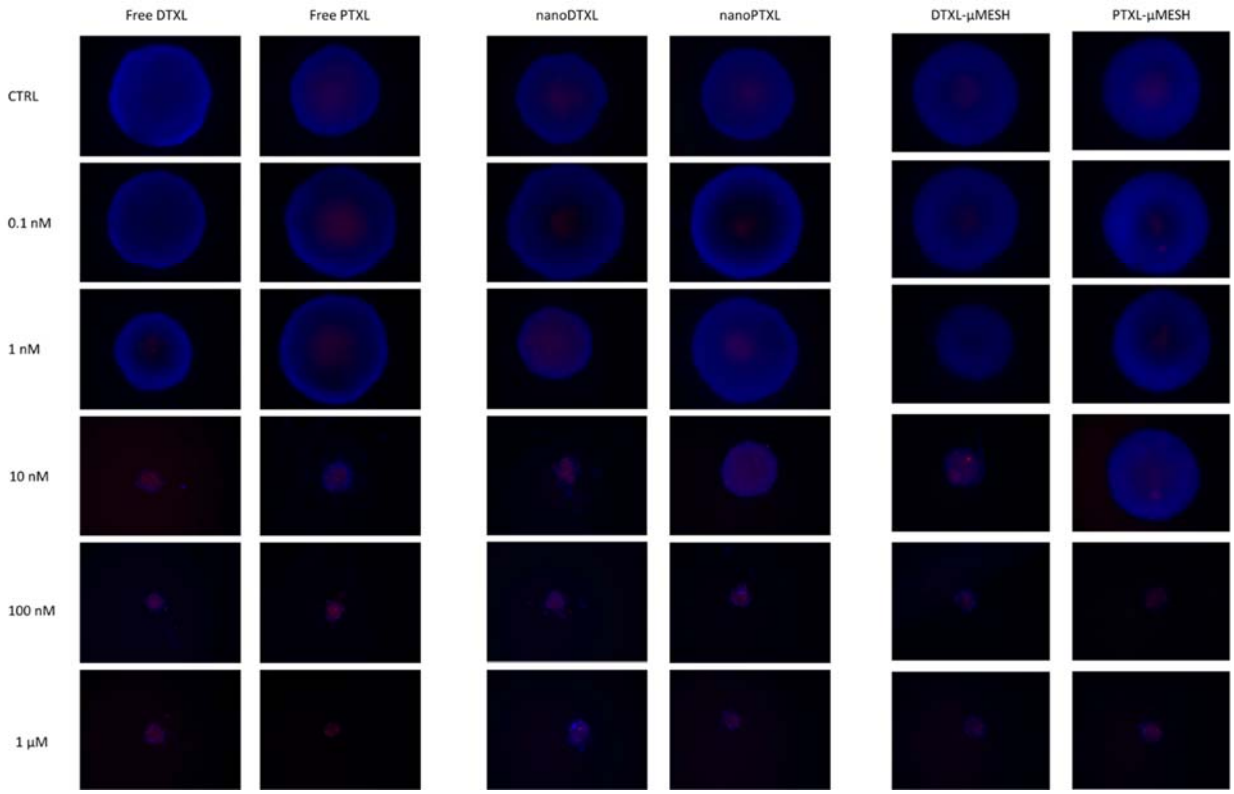

Supporting Figure 8. Cytostatic effect on U-87MG spheroids – tumor spheroids. Representative images comparing tumor spheroids exposed to different therapeutic regimens at day 10.

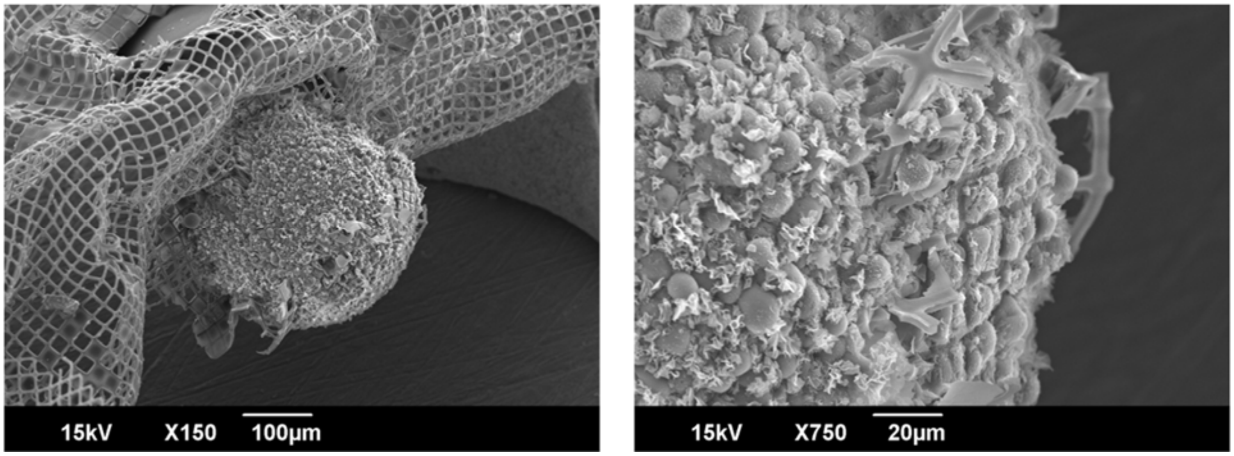

Supporting Figure 9. μMESH interaction with a tumor spheroid. Representative scanning electron images showing a μMESH wrapping around a tumor spheroid. An intimate interaction is established among the μMESH and the spheroid with cancer cells intercalating within the openings of the PLGA micronetwork, which carries the cytotoxic molecules.

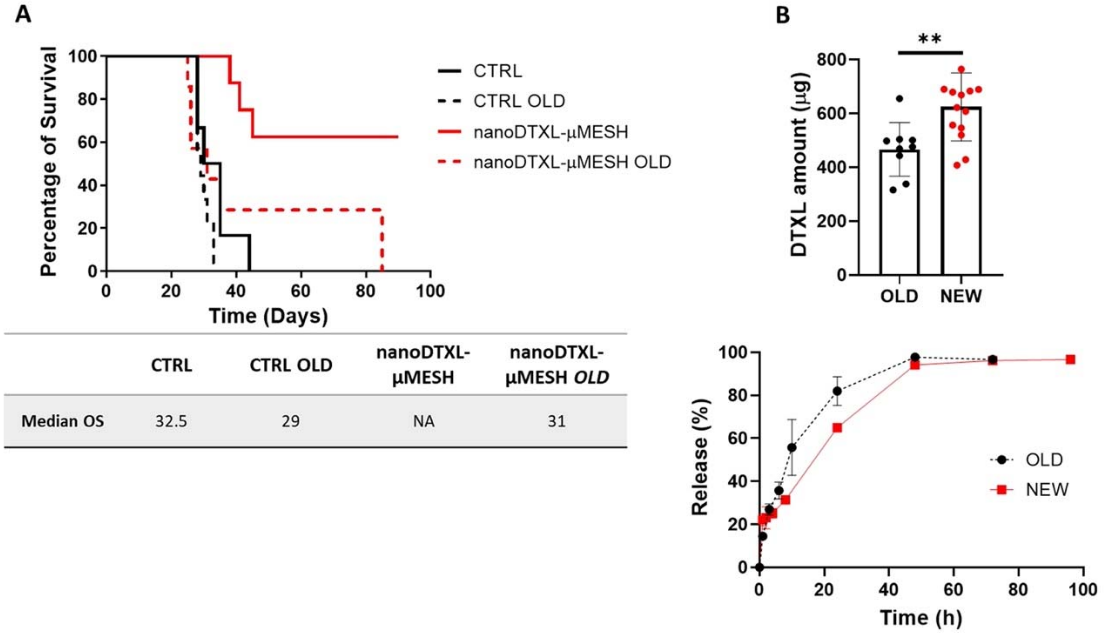

	CTRL	CTRL OLD	nanoDTXL-μMESH	nanoDTXL-μMESH *OLD*
Median OS	32.5	29	NA	31

Supporting Figure 10. Kaplan-Meier curves for glioblastomas treated with nanoDTXL. A. Direct comparison in terms of animal survival between a μMESH carrying nanoDTXL realized in this manuscript (continuous red line) and "nanoDTXL OLD" used in a previous work by the authors (dashed red lines).[1] **B.** The main difference between the two nanoDTXL formulations is in the total amount of docetaxel loaded per nanoparticle and the release dynamics. As such, upon release in the tumor parenchyma, the current nanoDTXL are expected to deploy a larger amount

of cytotoxic drug molecules per cancer cell and, therefore, produce a more prominent cytotoxic activity on the cells as compared to the previous work by the authors.

Supplementary References

(1) Di Mascolo, D.; Palange, A. L.; Primavera, R.; Macchi, F.; Catelani, T.; Piccardi, F.; Spano, R.; Ferreira, M.; Marotta, R.; Armirotti, A.; Gallotti, A. L.; Galli, R.; Wilson, C.; Grant, G. A.; Decuzzi, P. Conformable Hierarchically Engineered Polymeric Micromeshes Enabling Combinatorial Therapies in Brain Tumours. *Nat Nanotechnol* **2021**, *16* (7), 820-829. DOI: 10.1038/s41565-021-00879-3.
